# Chemical communication and its role in sexual selection across Animalia

**DOI:** 10.1038/s42003-023-05572-w

**Published:** 2023-11-20

**Authors:** Tyler J. Buchinger, Weiming Li

**Affiliations:** 1 Department of Fisheries and Wildlife, Michigan State University, East Lansing, MI USA; 2 Biology Department, Albion College, Albion, MI USA

**Keywords:** Sexual selection, Animal behaviour

## Abstract

Sexual selection has been studied as a major evolutionary driver of animal diversity for roughly 50 years. Much evidence indicates that competition for mates favors elaborate signaling traits. However, this evidence comes primarily from a few taxa, leaving sexual selection as a salient evolutionary force across Animalia largely untested. Here, we reviewed the evidence for sexual selection on communication across all animal phyla, classes, and orders with emphasis on chemoreception, the only sense shared across lifeforms. An exhaustive literature review documented evidence for sexual selection on chemosensory traits in 10 of 34 animal phyla and indications of sexual selection on chemosensory traits in an additional 13 phyla. Potential targets of sexual selection include structures and processes involved in production, delivery, and detection of chemical signals. Our review suggests sexual selection plays a widespread role in the evolution of communication and highlights the need for research that better reflects animal diversity.

## Abstract (web summary)

In this Perspective, the authors evaluate the evidence for sexual selection on communication across Animalia, with particular emphasis on chemosensory traits.

## Introduction

Animals interact with mates and sexual rivals using diverse and often elaborate traits^1^. These traits are among the most striking displays of animal biodiversity (e.g., courtship dance of peacock spider^2^) and inspired Darwin’s theory that sexual selection arising from variation in access to mates (or gametes)^3^ (Box 1) acts alongside selection for survival and fecundity^4^. The last few decades have brought an outpouring of research on the evolution of sexual ornaments, displays, and calls^5,6^ and, as Darwin suggested, overwhelming evidence indicates such traits often evolve under sexual selection. Having garnered empirical support as a salient evolutionary force underlying signaling traits^1^, sexual selection continues to hold the keen focus of evolutionary biologists due, in part, to the hypothesis that its effects on sexual signals and preferences drive reproductive isolation and ultimately speciation^7,8^. As important products of sexual selection and substrates for sexual selection to drive speciation, signaling traits are often at the center of discussions on the evolution of animal biodiversity^9–11^.

Despite its broad scope, theory around animal signals and sexual selection has advanced largely through the intensive study of relatively few taxa. Animals such as frogs, fish, and arthropods have proven particularly useful for developing and testing models of sexual selection^12,13^. For example, studies on túngara frogs^14^, Trinidadian guppies^15^, and fiddler crabs^16^ revealed that sexual signals can evolve to exploit receivers’ sensory ecology rather than providing information about the signaler’s quality as a mate^17^ (e.g., male guppies have orange spots that mimic fruit^18^). Largely lost amongst the mechanistic details of sexual selection, however, is the fundamental question of whether sexual selection acts as an important evolutionary force across all of Animalia. Importantly, all but one (Micrognathozoa) animal phyla include species that are known to reproduce sexually and therefore could be shaped by sexual selection^19^. The conspicuous signaling traits that originally captured Darwin’s attention clearly illustrate a major role of sexual selection in some chordate and arthropod classes^20^ but whether this role is common across the animal kingdom remains largely unexplored.

In this Perspective, we review the evidence for sexual selection on traits involved in chemical communication across the animal Tree of Life. As we outline below, chemical communication is uniquely poised to be a possible target of sexual selection across all animals and therefore particularly important for evaluating the potential for sexual selection on communication at a macroevolutionary scale. Our primary objective is to review the evidence for sexual selection on signaling traits across Animalia. By focusing on chemical communication, our approach explicitly acknowledges differences in animals’ sensory biology. After establishing chemosensation as the only possible target of sexual selection on communication that is common across all of Animalia, we review (1) the evidence for sexual selection on chemosensory traits in species from all phyla, classes, and orders of animals and (2) the mechanisms (e.g., mate choice; Table 1) and targets (e.g., scent glands; Table 2) of sexual selection on chemical communication. Our goal is to encourage the field of sexual selection to have a broader scope that spans Earth’s diverse forms of animal life, and advocate that chemical communication be a key focus of the discussion.

**Table 1:** Mechanisms of sexual selection on chemosensory traits (modified from refs. ^1,3^).

Mechanism	Example	Species
Scramble competition	Males with more sensilla on antennae locate females quicker^86^	Mantid (*Pseudomantis albofimbriata*)
Contest competition	Eventual winning males jet urine at opponents during fights to induce defensive behaviors^165^	Crayfish (*Astacus leptodactylus*)
Gamete competition	Love dart injects mucous into mates to delay remating^166^	Snail (*Euhadra quaesita*)
Mate choice	Males prefer pheromones of virgin over mated females^167^	Nematodes (*Caenorhabditis* sp.)
Cryptic mate choice	Females differ in the production of sperm chemoattractant and males differ in sperm chemotactic ability^168,169^	Urchin (*Lytechinus pictus*)

**Table 2:** Example targets of potential sexual selection on chemical communication.

Level	Trait type	Example	Species
Production	Enzyme	Female-specific expression of fatty acid elongase underlies sex pheromone production^110^	Cockroach (*Blattella germanica*)
	Cell	Male-specific gill cells release sex pheromone^81^	Lamprey (*Petromyzon marinus*)
	Gland	Male-specific leg glands secrete sex pheromone^116^	Velvet worm (*Cephalofovea tomahmontis*)
	Organ	Large kidneys and hypertrophic urinary bladders mediate pheromone signaling by dominant males^120^	Tilapia (*Oreochromis mossambicus*)
Delivery	Apparatus	Calcareous dart injects allohormone that biases paternity^170^	Snail (*Helix aspersa*)
	Signaling behavior	Dominant males kick feces to increase active space of odor^127,128^	Rhino (*Ceratotherium simum*)
Transmission	Accessory molecule	Proteins delay scent evaporation and extend signal duration^132^	Mouse (*Mus domesticus*)
Detection	Sampling behavior	Eventual losers of fights flick sensory antennules more often to assess urine signal of competitor^171^	Lobster (*Nephrops norvegicus*)
	Sensory structure	Long antennae improve male mate search^172^	Isopod (*Asellus aquaticus*)
	Accessory molecule	Male-specific binding proteins increases sensitivity to female pheromones^173^	Moth (*Chilo suppressalis*)
	Receptor	Female-specific expression of putative pheromone receptors on sensory tentacles^174^	Starfish (*Acanthaster planci*)
	Neural circuit	Sexually dimorphic neural circuit mediates sex-specific responses to a pheromone^175^	Fruit fly (*Drosophila melanogaster*)

Box 1 Glossary *Sexual selection*: Selection that arises from fitness differences associated with nonrandom success in the competition for access to gametes for fertilization^3^.

*Signal*: A trait of one individual (sender) that evolved to influence the physiology or behavior of another individual (receiver) after being sensed by the receiver^181^.

*Cue*: A sensory stimulus (biotic or abiotic) that triggers a response in an animal but did not evolve for communication^181^.

*Pheromone*: A molecule that evolved for signaling to conspecifics and elicit a specific reaction when detected^24^.

*Allohormone*: Molecules from one individual that are transferred directly into a conspecific and that elicit a physiological or behavioral response without being detected by external senses^134,182^.

### Chemosensory traits as potentially universal targets of sexual selection

Despite much interest in visual and auditory signals, most animals cannot see or hear. High-resolution, image-forming eyes are present only in arthropods, chordates, and cephalopod mollusks^21^, and only vertebrates and some arthropods possess ears or analogs of ears^22^. Although some animals that lack eyes or ears can detect light and sounds^21,23^, the sensitivity and specificity of these channels is unlikely sufficient for communication. For example, photoreception in most animal phyla can mediate internal physiological control (e.g., circadian rhythms), directional phototaxis, and habitat orientation but not interactions with specific objects or individuals (e.g., mates)^21^. In contrast, all single- and multicellular organism*s* have chemoreceptors that allow acute sensitivity to specific chemicals^24^.

The capacity to sense specific molecules is a fundamental feature of life on Earth^25,26^. Unicellular bacteria^27^, archaea^28^, protists^29^, and fungi^30^ express membrane-bound receptors that bind specific molecules, such as those related to social conditions (e.g., quorum-sensing pheromones)^31^. As first suggested by JBS Haldane, the external chemoreceptors of unicellular organisms may be precursors to internal receptors that allow intercellular communication in multicellular organisms^25^. This basic ability to detect chemicals in the milieu surrounding cells, whether internally or externally, has given rise to specialized chemosensory cells and organs in seemingly all animals, from the nerveless poriferans and placozoans to ctenophores and cnidarians, which have nerve nets, and the bilaterians with their centralized nervous systems^32,33^. The mechanisms of chemoreception differ within and among taxa, and include solitary chemosensory cells, olfaction, gustation, and the vomeronasal system. However, these classifications are largely based on terrestrial vertebrates and insects and may not hold in other taxa^34^, especially the many groups for which our understanding of chemoreception systems is limited (e.g., ctenophores)^35^. Regardless, the ubiquity of chemoreception as a specific sensory channel makes it especially useful for studying the potential role of sexual selection across diverse animals.

How and where animals live further implicates chemosensory traits as common potential targets of sexual selection across higher taxonomic levels. Sexual signals evolve under selection related to animals’ ecology—specifically how individuals interact with potential mates and the environment^15,36^. In several phyla, individuals that already lack vision and hearing also have limited ability to interact with mates via touch as they are sessile as adults^37^ (with some exceptions, such as sessile barnacles with extendable penises^38^). Perhaps more surprising to visually oriented humans is evidence that the dominant sensory environment of animals favors communication via chemoreception^39^; most invertebrates^40^ and mammals^41^ are nocturnal and a large proportion of Earth’s animal diversity is found in the perennial dark of the deep sea^42^ and underground^43^. Although many animals have adaptations that allow vision in dim light^44^, life in the dark is often associated with a predominant role of chemoreception^45–47^. Numerous and interacting ecological conditions shape the evolution of signaling systems and visual, auditory, electrical, and tactile communication clearly play dominant roles in many taxa^48^. However, the basic sensory capabilities and ecology of many animals suggest chemoreception is the only common channel for sexual communication across higher taxonomic levels of the animal Tree of Life.

### Taxonomic distribution of chemosensory traits potentially under sexual selection

#### Literature review

We searched for studies indicating that chemosensory traits guide sexual interactions between competitors or mates. Our primary objective was to document evidence for sexual chemosensory traits across higher taxonomic groups of Animalia (phyla *n* = 34, classes *n* = ~100, orders *n* = ~600). As the animal kingdom includes immensely diverse life-forms and studies on these life-forms often use different terminology, achieving our goal required a flexible and exhaustive search rather than a structured review with restricted search terms and filtering steps. We followed the taxonomy of Ruggiero et al.^49^ because it provided a unified classification down to the level of order and therefore allowed a taxonomically systematic search (Supplementary Data 1). To begin, we searched Google Scholar using both scientific and common names (when available), and keywords such as (but not limited to) “pheromone”, “chemical cue”, “chemosensory”, “olfactory”, and “scent”. At a minimum, we searched at the level of phyla and order. Searches for which the above keywords yielded few (or no) results were then repeated using more general keywords such as “reproductive behavior”, “mating”, and “spawning”. Often, these general searches yielded papers that were only tangentially relevant but either cited or were cited by studies that were directly relevant to our search. When available, we leveraged review papers^50–54^ and online resources (e.g., www.pherobase.com) that guided us to potentially relevant studies. In especially obscure taxa (e.g., Placozoans), we used Google Scholar or Google Search and no search terms other than their name to find if anything is known about how they reproduce and if chemosensory traits might be involved. Using these approaches, we searched exhaustively for the most direct evidence (see below for categories of evidence) of sexual selection on a chemosensory trait available for each order of animal. Importantly, our search was exhaustive in that we attempted to document, at a minimum, one example of a sexual chemosensory trait for each order but not exhaustive in compiling the available evidence within orders.

We included studies that fit into three categories according to the evidence they provided for sexual selection on a chemosensory trait. The first category met the criteria for sexual selection set by Andersson^1^: (i) evidence of a significant relationship between a trait and mating success and (ii) an identified mechanism of sexual selection, such as mate choice (Table 1). Consistent with Andersson^1^ and more recent literature^55^, this category included studies that used a proxy of mate choice (e.g., time near stimulus from a potential mate) but did not measure actual mating outcomes. To acknowledge known research biases towards certain taxa^12,13,56^, we also included studies in two additional categories if they indicated potential for sexual selection on a chemosensory trait but did not provide direct empirical support per the established criteria^1^. Specifically, studies considered to report potential for sexual selection on chemosensory traits included (i) documented behavioral or physiological responses to chemical traits of mates or competitors in a reproductive context (e.g., behavioral attraction of mature male Nautilus to female rectal extract^57^) or (ii) suggestions of sexual chemosensory interactions based upon indirect evidence (e.g., sexually dimorphic leg glands in centipedes^58^). We prioritized studies that fit in the first category, and sequentially included studies in the second and then third categories only if we were unable to find studies that met the criteria of the first category. Importantly, studies in the latter two categories do not provide direct evidence of sexual selection on chemosensory traits. Nevertheless, we included them to illustrate the state of the field across taxa and guide future work. Indeed, these relaxed criteria could produce an overestimate of the distribution of sexually selected chemosensory traits. For example, chemicals only documented to elicit responses in the opposite sex might turn out to guide sex or species recognition but not be shaped by sexual selection^20^ (but see ref. ^59^ regarding possible issues with the distinction between species recognition and mate choice).

Our review took a broad view of the chemosensory traits that guide sexual interactions. Although our primary focus was on signaling traits, we also included some chemosensory stimuli that may not fit classic definitions of signals (Box 1). In many cases, additional research is needed to determine whether a chemosensory stimulus is a signal that evolved for communication or a cue that elicits a response in receivers but did not evolve for that purpose. Indeed, traits detected by all sensory systems are often difficult to discretely categorize as cues versus signals^48^. For example, female goldfish (*Carassius auratus*) excrete hormonal metabolites via urine that attract males^60^; these molecules were initially considered cues that males spied on^61^, but subsequent research revealed females control their release of urine to facilitate communication^62^. In other cases, sexual chemosensory traits strain the classic definition of signals. For example, male salamanders (*Plethodon shermani*) release pheromone proteins that increase courtship receptivity after being smelled by females^63^; male frogs (*Rana temporaria*) produce closely related proteins suspected to also increase female receptivity but deliver them directly into females via spiny nuptial pads rather than releasing them into the environment^64^. Although the proteins transferred into female frogs may not fit classic definitions of a signal as they are not sensed as an external stimulus, they conceivably evolved via similar selective mechanisms and, in our opinion, are relevant to our review. We also noted studies on chemosensory traits that guide interactions between gametes (e.g., sperm chemotaxis) as they are similar targets of sexual selection in diverse animals^65^, though we only note these in our literature review if we were unable to find examples of chemosensory traits that guide interactions between individuals. Lastly, we did not distinguish between the various mechanisms involved in the detection of chemical traits (e.g., olfaction versus taste)^24^. As discussed above, a flexible and inclusive approach was necessary to support a discussion on sexual selection across the diversity of Animalia.

#### Potential for sexual selection on chemosensory traits across Animalia

**Fig. 1:**
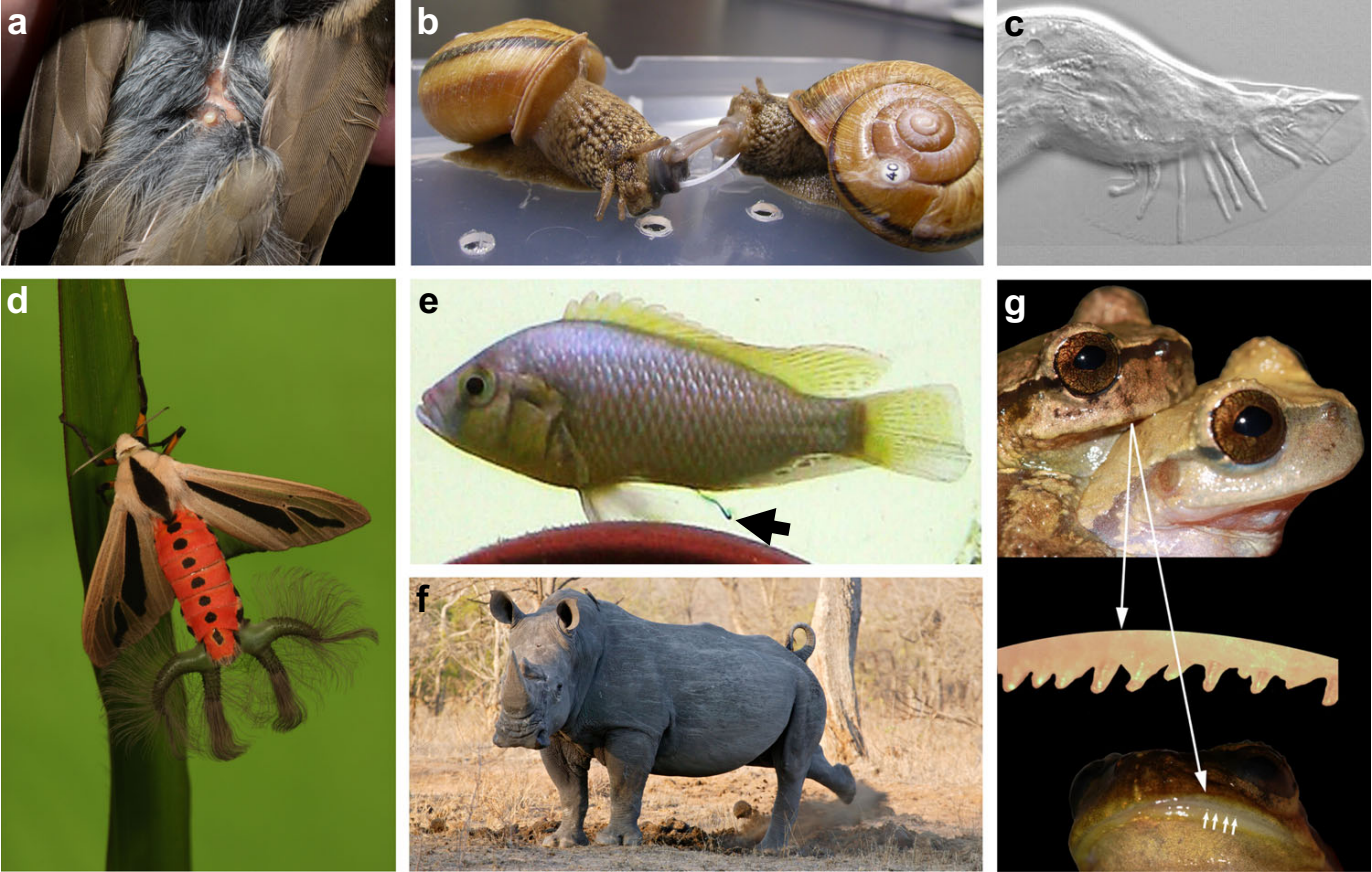
Examples of chemosensory traits potentially under sexual selection in animals. **a** Preen gland involved in olfactory signaling in birds (Swainson’s thrush, *Catharus ustulatus*; photo credit: Brock and Sherri Fenton). **b** love dart of land snail (*Bradybaena pellucida*) that injects allohormone into mate to increase paternity (photo credit: Kazuki Kimura). **c** sensory rays of male nematode (*Caenorhabditis elegans*) used in chemosensation of mates (image adapted with permission from ref. ^176^). **d** inflatable scent gland of male tiger moths (*Creatonotus gangis*; photo credit: Darren5907/Alamy). **e** pulse of urine released by dominant male cichlids (*Astatotilapia burtoni*; image adapted with permission from ref. ^177^). **f** Male white rhino (*Ceratotherium simum*) kicking dung to spread chemical signal (AfriPics.com/Alamy). **g** Protruding teeth and swollen lips in frogs (*Plectrohyla sagorum*) that males use to scratch females and deliver putative pheromones (reproduced from ref. ^137^, BMC).

**Fig. 2:**
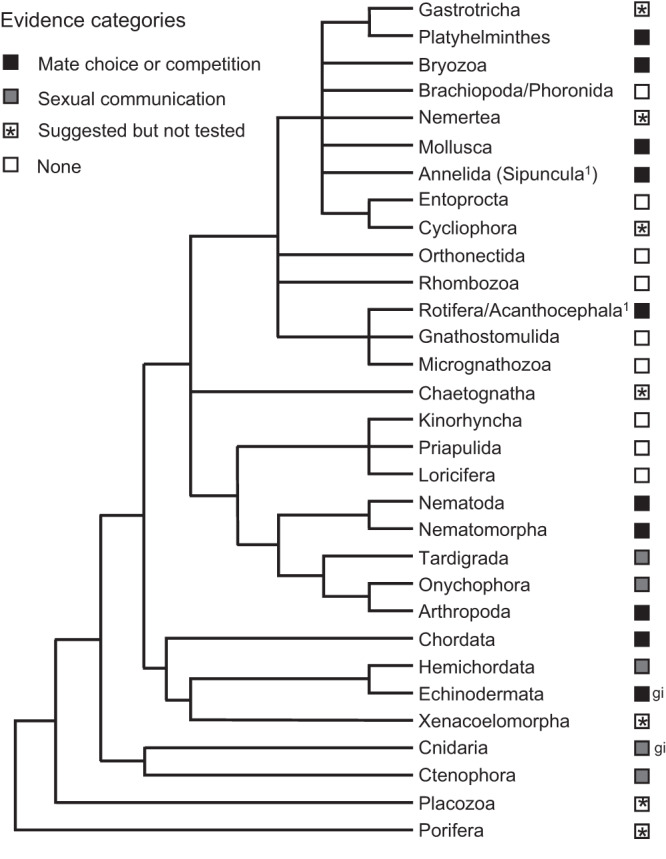
Evidence and potential for sexual selection on chemosensory traits across animal phyla. Black-filled boxes indicate phyla for which evidence suggests chemosensory traits guide mate choice or competition. Gray-filled boxes indicate phyla for which chemosensory interactions between mates or sexual rivals have been documented. Asterisks indicate phyla for which chemosensory interactions between mates or sexual rivals have been suggested but remain without direct empirical support. *gi* indicates the trait with the strongest support in a phylum was involved in gamete interactions. Phylogeny based on ref. ^178^. ^1^Sipuncula and Acanthocephala were considered distinct phyla in the taxonomy followed for our literature review^49^ but are recognized in ref. ^178^ as part of Annelida and Rotifera. Sexual chemical communication has been documented in Acanthocephala but remains untested in Sipuncula.

Potential for sexual selection on chemosensory traits spans across the Tree of Life (Figs. 1 and 2 and Supplementary Data 1 and 2). Altogether, our literature review included *n* = 319 studies on the potential for sexual selection on chemosensory traits. Studies on animals from 10 of 34 phyla provide evidence of sexual selection on chemosensory traits that meets the criteria set by Andersson^1^. Among these phyla, 9 include animals with traits involved in communication (e.g., chemosensory-based mate preferences) and 1 (Echinodermata) includes animals with traits that guide gamete interactions (cryptic mate choice). An additional 6 phyla possess chemosensory traits that were found to guide interactions between mates or competitors (5 at the individual level, 1 at the gamete level), and therefore may be under sexual selection. However, a direct link between variation in these traits and mating success has yet to be established. Finally, sexual chemosensory traits have been suggested for 7 more phyla, though direct evidence for sexual communication remains lacking. Although our review focused on the animal kingdom, fungi^66^, bacteria^67^, protists^68^, and plants^69^ also have reproductive chemosensory traits that may be shaped by sexual selection. Importantly, our failure to find evidence for sexual selection on chemosensory traits in many taxa should not be interpreted as evidence against sexual selection in those taxa. Indeed, the literature shows evidence (Category 1) or potential indications (Category 2 or 3) of sexual selection on chemosensory traits across the Tree of Life and in most animal phyla (23 of 34).

**Fig. 3:**
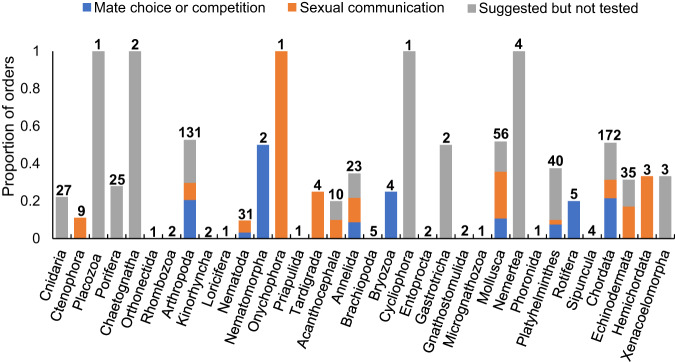
Proportion of orders with evidence or potential for sexual selection on chemosensory traits across phyla. Studies are categorized according to whether they (1) report evidence that chemosensory traits guide mate choice or competition, (2) report evidence for sexual chemical communication, or (3) suggest sexual chemical communication without direct evidence. Numbers above bars indicate the number of orders within each phyla, based upon ref. ^49^. For clarity, only traits that mediate interactions between mates or competitors (not gametes) are included, though cryptic mate choice via gamete chemosensation has been shown or suggested in many orders of Echinodermata and Cnidaria (Supplementary Data 1).

Albeit relatively broad, the taxonomic distribution of evidence for sexual selection on chemosensory traits remains shallow, with most phyla represented by relatively few classes or orders (Fig. 3 and Supplementary Data 1). In phyla with chemosensory traits that meet the criteria for sexual selection (Category 1), only Annelida, Bryozoa, Nematomorpha, and Platyhelminthes had evidence from more than half of classes, and these four phyla each have <3 recognized classes. However, most classes (≥50%) in phyla Cnidaria, Ctenophora, Porifera, Arthropoda, Nematoda, Tardigrada, Annelida, Bryozoa, Mollusca, Nemertea, Platyhelminthes, Rotifera, Chordata, Echinodermata, and Hemichordata have at least some potential indications (Category 2 or 3) of sexual selection on chemosensory traits, even if the indications are only suggestions based on indirect evidence. Phyla Placozoa, Chaetognatha, Nematomorpha, Onychophora, Cycliophora, and Gastrotricha showed potential indications of sexually selected chemosensory traits but have ≤1 recognized classes (see ref. ^49^; Supplementary Data 1), making the percentage of represented classes impossible or of little use to calculate. Finally, Acanthocephala and Xenacoelomorpha had indications of sexually selected chemosensory traits in <50% of classes.

In many taxa, the lack of evidence for sexual selection on chemosensory traits likely represents a lack of data rather than true absence. Indeed, the animal kingdom is rife with poorly understood life-forms. Phyla Loricifera, Cycliophora, and Micrognathozoa were only discovered in the last several decades^70–72^. Furthermore, sexual reproduction has not yet been confirmed in Micrognathozoa^73^ and was only recently documented in Placozoa^74^. The monotypic cnidarian *Polypodium hydriforme*, a parasite found only in eggs of a small order of fishes (Acipenseriformes), may be exclusively parthenogenetic^75^ and therefore not subject to sexual selection^19^. Coelacanths, one of only five classes of chordates for which indications of sexual selection on chemosensory traits are lacking, were thought long extinct before their rediscovery in 1938^76^; today, basic questions about their reproductive behavior, such as how they interact with mates, remain unanswered^77^. Improving our basic understanding of some clades will almost certainly unveil more evidence of sexual selection on chemosensory traits.

### Potential targets of sexual selection on chemosensory traits across Animalia

**Fig. 4:**
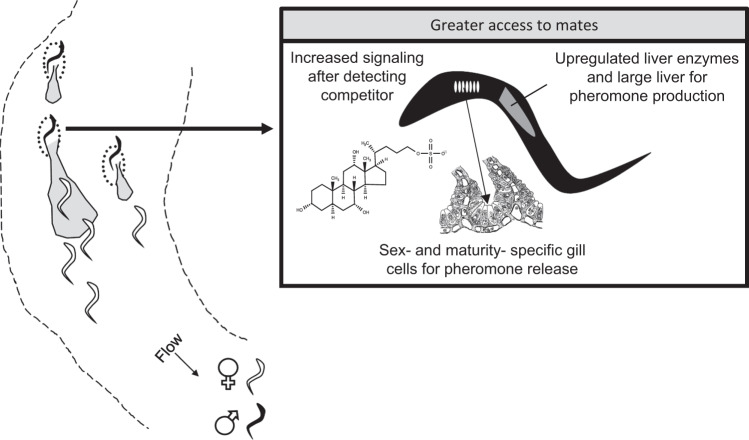
Illustration of how sexual selection can act on various traits underlying sexual chemosensory interactions. During spawning, female sea lamprey (*Petromyzon marinus*) orient towards the bile acid 3-keto petromyzonol sulfate (3kPZS). The lek-like mating system of lamprey and female preference for the more concentrated of adjacent 3kPZS plumes^179^ appears to generate sexual selection for upregulation of bile acid synthetic enzymes^79,84^, large livers (where bile acids are biosynthesized)^80^, and sexually dimorphic gill cells involved in 3kPZS release^81^. Furthermore, males increase the attractiveness of their pheromone signal by releasing more 3kPZS after exposure to a competitor^83^. Gill cell image adapted with permission from ref. ^180^.

Sexual selection acts upon a diverse collection of chemosensory traits spanning molecules to behaviors (Table 2). Examining the specific targets of sexual selection is helpful for two primary reasons:

First, it shows where signatures of sexual selection might occur. By definition, sexually selected chemosensory traits influence individuals’ success at accessing mates or gametes^3^. This influence on mating success arises from various traits at all levels of biological organization (e.g., from cells to behaviors), not just the signal or sensory structure directly involved in sexual interactions^78^. For example, female preference for higher pheromone concentrations favors higher signaling rates in sea lamprey (*Petromyzon marinus*), but various physiological^79–81^ and behavioral traits^82,83^ that mediate pheromone production and release are likely the specific targets of sexual selection (Fig. 4). In lampreys, the traits underlying pheromone signaling show possible signatures of sexual selection, such as sexual dimorphism^81^ and relatively high inter-specific diversity^84^. Studying the various traits underlying chemical communication can be especially useful for testing potential sexual selection when the specific identity of the signal is unknown.

Second, examining the targets of sexual selection can reveal the various mechanisms by which sexual selection can act upon communication across diverse taxa. Theory suggests that sexual selection acts on communication traits via several mechanisms of mate choice and competition^3,6^ (Table 1). Much evidence supports this theory, but the data come from relatively few animal groups^1^. For chemical communication, mate choice using signals that provide or indicate benefits to the choosing sex has been of particular interest^85^; however, other mechanisms (Table 1) of sexual selection are also important and may be more relevant for some animal groups. For example, large and elaborate sensory structures in male insects indicate a possible role of sexual selection via scramble competition (discussed below^86^). Indeed, most studies focus on signals that attract mates or repel competitors^20^ but such functions do not fully capture the diversity of sexual chemosensory traits. Below, we review the potential targets of sexual selection on chemical communication and the mechanisms by which sexual selection can act on each.

#### Molecular constituents

Animals use a diverse selection of molecules to interact with mates and sexual rivals^24^. Chemical diversity in these traits arises from differences in attributes of individual compounds, such as their class (e.g., protein vs. steroid vs. ketone)^87–89^, functional group^90^, or stereochemistry^91^, as well as differences in mixture constituents or proportions^92^. Even different concentrations of a single compound can be perceived as distinct stimuli^93^. High species specificity of many sexual chemical signals indicates they often diversify rapidly^94^. This diversity has made chemical signals an especially useful model of studying the evolution of communication^94^. However, the classical view on how chemical signals evolve emphasized species recognition^95^ and only more recently has the scope broadened to include sexual selection^85,96^. Nevertheless, a substantial body of evidence, largely but not exclusively from studies on insects^97^, shows that sexual selection can influence the molecular identity of chemical signals.

Sexual selection can act through several mechanisms to influence the identity of chemical signals. In some cases, the molecular constituents of chemical signals are closely linked to the benefits the signal provides or indicates to receivers. Insect pheromones are often molecules sequestered from needed resources^85^, such as the plant-derived alkaloids that deter predators and act as male sex pheromones in the moth *Utetheisa ornatrix*^98,99^. In fish, hormonal metabolites directly indicate the reproductive status of the signaler^60^ and peptides associated with major histocompatibility complex (MHC) molecules reflect genetic quality^100^. Signals can also evolve to exploit pre-existing aspects of receivers’ sensory biology without necessarily providing any benefit^18^, though evidence for this evolutionary mechanism of mate choice is not well-documented for chemical communication^101^. A few examples include prey molecules released by male beewolfs (*Philanthus triangulum*) and rock lizards (*Iberolacerta cyreni*) to attract conspecific females searching for food^102,103^ and a bile acid released by male sea lamprey to mimic a larval cue used to navigate to preferred habitats^104^. Though the above studies focus on interactions between mates, similar mechanisms act upon chemical signaling between rivals; male goldfish (*Carassius auratus*) mediate aggressive interactions using reproductive hormones that are likely related to their reproductive status^105,106^ and male *Drosophila* manipulate competitors using an anti-aphrodisiac pheromone that exploits a pre-existing sensory bias^107^. Finally, sexual selection might also drive elaboration of chemical signals if adding components increases signal information content or efficiency^92,94,97^, though evidence that this occurs also remains limited^108^. Unfortunately, a poor understanding of the specific chemical structures of chemical signals in most animals limits broad inferences about the link between their molecular composition and the mechanisms of sexual selection that act upon them^94^.

#### Traits related to the production of chemical signals

Chemical signals manifest through an assortment of molecular and physiological processes, cells, and organs that are shaped by sexual selection^109^. Often, sexual chemical signals are produced via sex- and stage-specific upregulation of biosynthetic enzymes or transporters^79,110^. These molecular processes can occur in cells with other functions or in sexually dimorphic cells that likely evolved to support chemosensory interactions between mates or sexual rivals^81,111^. Similarly, cells involved in chemical signaling can be dispersed throughout the body, localized to common organs, or organized into specialized glandular tissues^109^. Pheromone and scent glands likely shaped by sexual selection are often noted in insects and mammals^24^ but also occur in fish^112^, birds^113^, anurans^114^, reptiles^115^, non-insect arthropods (centipedes)^58^, onychophorans^116^, nemerteans^117^, gastrotrichs^118^, and platyhelminths^119^. Common organs can also have adaptations for producing or emitting sexual chemical signals (e.g., large livers^80^, hypertrophic urinary bladders^120^). Adaptations for sexual signaling in common organs, albeit more cryptic than those associated with specialized glands, may be especially widespread given many chemical signals are released via routes linked to common physiological processes (e.g., feces, urine)^24^.

#### Traits related to the delivery of chemical signals

Sexual selection can act upon physiological and behavioral traits that mediate delivery of chemical signals. Many chemical signals consist of molecules that also have non-communicative functions^92^ and leak out via sexual materials^121^, tears^122^, mucous^123^, feces^124^, urine^125^, and respiratory waste^126^. Release via seemingly unspecialized routes again points to selection for signals with direct links to the physiological status of signalers. As discussed above, chemicals that leak out may act as cues that receivers evolved to detect but not signals that involve any adaptations in releasers (Box 1). However, even unspecialized routes of release often involve finesse that evolved for communication; for example, dominant male white rhinos (*Ceratotherium simum*) defecate more often than females or nonterritorial males and kick their dung to increase the signal’s active space^127,128^. Controlling when and where to signal may allow signalers to deceive receivers with chemical signals that seem otherwise difficult to fake^129^. Alternatively, tactical signal delivery may arise via selection for signal efficiency^15,130^. For example, male swordtails (*Xiphophorus birchmanni*) urinate more often in the presence of females and orient themselves upstream of females when courting, presumably to help deliver chemical signals^131^. Analogous non-behavioral traits can also facilitate signal delivery; some animals produce proteins that bind chemical signals (e.g., major urinary proteins in mice) to (1) slow evaporation of the molecule, thereby extending signal duration^132^ or (2) release the chemical only upon arrival to the sensory organ according to its local chemical environment (e.g., pH)^133^.

Some sexual chemicals are delivered directly into the body without being detected by receiver’s external sensory systems (allohormones; Box 1)^134^. For example, males in some plethodontid salamanders open females’ skin using hypertrophic teeth and then rub their mental gland on the wound to inject directly into the blood a pheromone that increases female receptivity^78^. In addition to injection through skin using various methods^135–137^, sexual chemicals can be delivered directly through insemination^138^ or consumption via nuptial gifts^139^. Importantly, chemical traits delivered directly into receivers’ bodies are generally not considered signals, which are detected by receivers’ sensory systems^134,140^. However, the line between these chemicals and conventional signals can be blurry, especially in closely related species that use the same class of molecules to interact with mates but differ in whether they deliver the chemicals to sensory systems or directly into the body^78^. Regardless, chemicals that bypass sensory systems are important targets of sexual selection^134^. Across various animal phyla, including commonly studied arthropods^141^ but also hermaphroditic annelids, platyhelminths, and mollusks^135,136,142,143^, chemicals directly transferred to females prevent digestion or disposal of sperm, suppress future mating, and ultimately bias paternity. In an interesting twist, chemicals transferred to mates can subsequently be or modify chemical signals for competitors^144^; for example, male moths mark females with an anti-aphrodisiac that deters other males^145^ and inject females with substances that inhibit them from producing pheromones that attract males^146^. Post-copulatory gamete competition^135,147^ and sexual conflict^136,148^ are usually suggested as sources of selection on these traits, but in some cases signal efficiency^137^ and mate choice may also play a role^134^.

#### Traits related to the detection of chemical signals

Animals detect chemical signals using a series of molecular, physiological, and behavioral traits that are often sexually dimorphic. Darwin hypothesized that sexually dimorphic sensory capabilities arise via sexual selection when males bear the primary burden of mate search^4,149^. In the 150 years since, research on sexual selection has focused more on signals and attributes of receiver sensory systems that influence the evolution of signals. Nevertheless, sexual dimorphism has been reported in many taxa and for nearly all levels of chemosensory detection (Table 2), ranging from the behaviors involved in sampling chemical stimuli^150^ to the neural circuits involved in processing chemical stimuli^151,152^. Importantly, sexual dimorphism in sensory traits can arise from differences in the ecology or biology of males and females rather than competition for mates^149,153,154^. However, empirical evidence, especially from arthropods, supports Darwin’s hypothesis that sexual selection via scramble competition favors greater chemosensory capacity in males^86,155–158^. Similar selection on detection of chemicals from potential mates may also be important in broadcast spawners, some of which release gametes after exposure to chemicals in the sexual fluids of mates or competitors^159^ and have higher reproductive success when given the first opportunity for fertilization^160^. Interestingly, sexual selection may also act on chemosensory capabilities via mate choice; in moths, females may choose high-quality mates by releasing minute quantities of pheromone only detectable by males with the most sensitive olfactory systems^149^. Sexual selection on chemosensory detection traits is less studied than sexual selection on chemical signals but may be especially important in many animals that use chemical information during scramble competition for mates or fertilizations^157^.

## Conclusions

Decades of empirical and theoretical research have focused on sexual selection as a major evolutionary driver of animal biodiversity. Signaling traits have been at the center of this work, as they are diverse, often appear extreme, and can lead to speciation when divergent preferences generate assortative mating. However, sexual selection as a broad and versatile evolutionary force across higher taxonomic levels of animals remains surprisingly undertested as most studies on sexual selection^12,13^, animal behavior^56^, and chemical communication^94^ focus on very few clades. Furthermore, most research on sexual selection has focused on communication via vision and hearing, which most animal phyla lack. In this Perspective, we reviewed the evidence for sexual selection on signaling traits across Animalia as a whole, with particular emphasis on chemosensory traits. Our review illustrates two especially important and related points, which we discuss below.

First, the broad scope of theory around sexual selection and animal signals stands in sharp contrast to the limited higher-level taxonomic distribution of supporting empirical studies. Clearly, extensive evidence supports sexual selection as a powerful evolutionary force on signaling traits in many species within a few clades^1,5,6^, especially some arthropods and chordates^12,13,56^. After searching for evidence of sexual selection on chemosensory traits across all animal phyla, classes, and orders, we found studies that meet established criteria of sexual selection^1^ in 10 of 34 animal phyla, and studies that report possible indications of sexual selection on chemosensory traits in an additional 13 phyla. Despite the clear potential for sexual selection on chemical signaling traits across diverse taxa, additional work is needed even in taxa for which current evidence meets established criteria of sexual selection^1^. Foremost is a basic need for more direct tests of sexual selection in most taxa; many studies we found only scratched the surface of how chemosensory traits could affect mate choice or competition. Even when traits clearly affect mate choice or competition, the actual strength of sexual selection on them depends on various deterministic (e.g., operational sex ratio) and random processes that underlie variation in mating success^161^. Our review indicates sexual selection could play a common role in the evolution of chemical communication but highlights the need for research that better reflects the diversity of animals (see Box 2).

The need for research that better represents all animals raises our second major point: chemosensory traits are arguably the primary (potential) target of sexual selection on communication when considering Animalia as a whole. A basic implication of this point is that chemosensation should be a key focus of research on sexual selection. This will ensure we do not underestimate the role of sexual selection in Animalia or predicate concepts about the prevailing mechanisms or consequences of sexual selection on sensory systems that may not be representative of many animals. Unfortunately, chemoreception is also among the least studied channels of communication^12^, due, in part, to the challenge of identifying the molecules that make up signals^101^ and the cryptic nature of most chemical signals^162^. Human biases further inhibit research on chemoreception^162^, and are exemplified by the superlatives used to characterize the traits that inspired the theory of sexual selection and continue to hold the attention of evolutionary biologists; what is an ‘extreme’ or ‘striking’ chemical signal? Emphasis on chemosensation is key to understanding sexual selection as a potentially universal and potent evolutionary force on animal communication.

Many interesting questions about sexual selection on animal communication remain unanswered^20,163^. We suggest one of the most fundamental of these is whether sexual selection acts as a salient evolutionary force on communication across Animalia. Admittedly, determining when this question has been answered is challenging. Nevertheless, pursuing the answer will reveal if and how the mechanisms and consequences of sexual selection differ across animals. This information is critical to develop a fuller understanding of how Earth’s animal diversity arose and to conserve this diversity in the face of rapid global change^164^.

Box 2 Looking forward *Recommendations*. We offer several recommendations that could help the study of sexual selection on communication to better span across Animalia.

1. *Increase taxonomic diversity in studies of sexual selection on communication*. Prioritizing taxa for future study could depend on the specific research questions being addressed, but we hope our literature review (Suppl. Data 1) will be a useful guide for identifying key knowledge gaps.
2. *Consider the many potential targets of sexual selection*. The molecular, physiological, and behavioral scaffolding underlying chemical communication can be particularly useful for studying sexual selection^183^ when the identity of a signal remains unknown or when the mating behavior of a species is poorly understood.
3. *Leverage new and developing techniques*. New and developing technology will likely accelerate research on chemical signals. For example, metabolomics—the global analysis of small molecules—shows promise as a powerful tool to study the molecular basis of chemosensory interactions^184^. Similarly, we expect other omic techniques (genomics, transcriptomics) will shed new light on the molecular and physiological processes underlying sexual selection on chemical communication.
4. *Expand the community of researchers studying sexual selection*. Achieving wider taxonomic representation is likely to rely, in large part, on researchers with specific taxonomic or technological expertise who currently focus on other research questions. We call for a broader community of scientists to study sexual selection on communication, and especially invite the attention of biologists studying animals that are not well represented in the field (see Figs. 2 and 3, Supplementary Data 1).

*Future directions*. Better representation across Animalia will support macroevolutionary studies that can test fundamental hypotheses about sexual selection. For example, Wiens and Tuschhoff^20^ provide an insightful discussion on how macroevolutionary studies could help explain the diversity of sexually selected signals and weapons. Below, we outline a few additional examples:

1. *Sexual selection and diversification of chemosensory receptors*. Chemoreceptors are encoded by some of the fastest evolving and largest gene families in the metazoan genome^185^ and in some species evolved under sexual selection^186,187^. Although chemoreceptor repertoires are well-characterized primarily in vertebrates and insects^188^, this list is likely to grow rapidly with the number of animal genomes sequenced^189–194^, putting in reach an understanding of how sexual selection shapes receiver evolution at a molecular level but macroevolutionary scale.
2. *Sexual selection and nervous system evolution*. The brain is a key player in the process of sexual selection and, for example, complicates sexual decisions by integrating inputs related to nonsexual tasks^195^. However, animals differ considerably in the complexity of their nervous systems, both among^196^ and within phyla^197^. How does the role of sexual selection on communication track the evolution of the nervous system in animals?
3. *Sexual selection and speciation*. The role of sexual selection in speciation has been the focus of a vibrant discussion for decades^7–11^. However, empirical tests of speciation by sexual selection have focused largely on arthropods and chordates^9^, which are among the most taxonomically rich phyla^198^. Greater taxonomic diversity in research on sexual selection will likely enrich discussions on the role of sexual selection in speciation.

### Supplementary information

Description of Supplementary Materials

Supplementary Data 1

Supplementary Data 2

## Data Availability

Our paper presents no new data. The results of the literature review are provided as Supplementary data.
