# Supplementary material for: Chemical communication and its role in sexual selection across Animalia
Source: Commun Biol. 2023 Nov 20;6:1178. doi: 10.1038/s42003-023-05572-w (PMC10662023; doi:10.1038/s42003-023-05572-w)
Supplement: Supplementary file 1 — Description of Supplementary Materials [file 42003_2023_5572_MOESM1_ESM.docx]

**Description of Additional Supplementary Files**

**File name:** Supplementary Data 1

**Description:** Results of the literature review

**File name:** Supplementary Data 2

**Description:** References for Suppl. Data 1
