# Supplementary material for: Chemical communication and its role in sexual selection across Animalia
Source: Commun Biol. 2023 Nov 20;6:1178. doi: 10.1038/s42003-023-05572-w (PMC10662023; doi:10.1038/s42003-023-05572-w)
Supplement: Supplementary file 3 — Supplementary Data 2 [file 42003_2023_5572_MOESM3_ESM.docx]

**Suppl. Data 2. References for Suppl. Data 1**

1 Adamo, S. A. & Chase, R. The “love dart” of the snail Helix aspersa injects a pheromone that decreases courtship duration. *J. Exp. Zool.* **255**, 80-87 (1990).

2 Addesso, K. M., Short, K. A., Moore, A. J. & Miller, C. W. Context-dependent female mate preferences in leaf-footed cactus bugs. *Behaviour* **151**, 479-492 (2014).

3 Amcoff, M., Hallsson, L. R., Winberg, S. & Kolm, N. Male courtship pheromones affect female behaviour in the swordtail characin (Corynopoma riisei). *Ethology* **120**, 463-470 (2014).

4 Ayasse, M., Engels, W., Lübke, G., Taghizadeh, T. & Francke, W. Mating expenditures reduced via female sex pheromone modulation in the primitively eusocial halictine bee, Lasioglossum (Evylaeus) malachurum (Hymenoptera: Halictidae). *Behav. Ecol. Sociobiol.* **45**, 95-106 (1999).

5 Bahr, A., Sommer, S., Mattle, B. & Wilson, A. B. Mutual mate choice in the potbellied seahorse (Hippocampus abdominalis). *Behav. Ecol.* **23**, 869-878 (2012).

6 Balthazart, J. & Schoffeniels, E. Pheromones are involved in the control of sexual behaviour in birds. *Naturwissenschaften* **66**, 55-56 (1979).

7 Barata, E. N., Hubbard, P. C., Almeida, O. G., Miranda, A. & Canário, A. V. Male urine signals social rank in the Mozambique tilapia (Oreochromis mossambicus). *BMC Biol.* **5**, 1-11 (2007).

8 Barata, E. N. *et al.* Putative pheromones from the anal glands of male blennies attract females and enhance male reproductive success. *Anim. Behav.* **75**, 379-389 (2008).

9 Barnes, H., Barnes, M. & Klepal, W. Studies on the reproduction of cirripedes. I. Introduction: copulation, release of oocytes, and formation of the egg lamellae. *J. Exp. Mar. Biol. Ecol.* **27**, 195-218 (1977).

10 Barry, K. L. Influence of female nutritional status on mating dynamics in a sexually cannibalistic praying mantid. *Anim. Behav.* **80**, 405-411 (2010).

11 Bartel, S. & Hohberg, K. Experimental investigations on the partner-finding behaviour of Isohypsibius dastychi (Isohypsibiidae: Tardigrada). *Zool. J. Linn. Soc.* **188**, 878-886 (2020).

12 Basil, J. A., Lazenby, G. B., Nakanuku, L. & Hanlon, R. T. Female Nautilus are attracted to male conspecific odor. *Bull. Mar. Sci.* **70**, 217-225 (2002).

13 Beauché, F. & Richard, F.-J. The best timing of mate search in Armadillidium vulgare (Isopoda, Oniscidea). *PloS One* **8**, e57737 (2013).

14 Berglund, A. Sequential hermaphroditism and the size-advantage hypothesis: an experimental test. *Anim. Behav.* **39**, 426-433 (1990).

15 Bills, M. L. *Description of the Chemical Senses of the Florida manatee, Trichechus manatus latirostris, in Relation to Reproduction*, Ph.D Dissertation, University of Florida, (2011).

16 Bishop, J. D., Manriquez, P. H. & Hughes, R. N. Water–borne sperm trigger vitellogenic egg growth in two sessile marine invertebrates. *Proc. R. Soc. B: Biol. Sci.* **267**, 1165-1169 (2000).

17 Boal, J. G. Female choice of males in cuttlefish (Mollusca: Cephalopoda). *Behaviour* **134**, 975-988 (1997).

18 Bohnet, S., Rogers, L., Sasaki, G. & Kolattukudy, P. Estradiol induces proliferation of peroxisome-like microbodies and the production of 3-hydroxy fatty acid diesters, the female pheromones, in the uropygial glands of male and female mallards. *J. Biolog. Chem.* **266**, 9795-9804 (1991).

19 Bone, L. W. Reproductive chemical communication of helminths. I. Platyhelminthes. *Int. J. Invert. Reprod.* **5**, 261-268 (1982).

20 Bone, L. W. Reproductive chemical communication of helminths. II. Aschelminthes. *Int. J. Invert. Reprod.* **5**, 311-321 (1982).

21 Borowsky, B. Effects of receptive females' secretion on some male reproductive behaviors in the amphipod crustacean Microdeutopus gryllotalpa. *Mar. Biol.* **84**, 183-187 (1984).

22 Breed, M. D., Smith, S. K. & Gall, B. G. Systems of mate selection in a cockroach species with male dominance hierarchies. *Anim. Behav.* **28**, 130-134 (1980).

23 Breithaupt, T. & Eger, P. Urine makes the difference: chemical communication in fighting crayfish made visible. *J. Exp. Biol.* **205**, 1221-1231 (2002).

24 Bryant, B. P. & Atema, J. Diet manipulation affects social behavior of catfish. *J. Chem. Ecol.* **13**, 1645-1661 (1987).

25 Buresch, K. C. *et al.* Contact chemosensory cues in egg bundles elicit male–male agonistic conflicts in the squid Loligo pealeii. *J. Chem. Ecol.* **29**, 547-560 (2003).

26 Caldwell, R. L. Assessment Strategies in Stoma Topods. *Bull. Mar. Sci.* **41**, 135-150 (1987).

27 Carazo, P., Sanchez, E., Font, E. & Desfilis, E. Chemosensory cues allow male Tenebrio molitor beetles to assess the reproductive status of potential mates. *Anim. Behav.* **68**, 123-129 (2004).

28 Carey, C. & Bull, C. Recognition of Mates in the Portuguese Millipede, Ommatoiulus-Moreletii. *Aust. J. Zool.* **34**, 837-842 (1986).

29 Carolsfeld, J., Tester, M., Kreiberg, H. & Sherwood, N. Pheromone-induced spawning of Pacific herring. *Horm. Behav.* **31**, 256-268 (1997).

30 Cartolano, M. C., Babcock, E. A. & McDonald, M. D. Evidence that Gulf toadfish use pulsatile urea excretion to communicate social status. *Physiol. Behav.* **227**, 113182 (2020).

31 Cartolano, M. C., Tullis-Joyce, P., Kubicki, K. & McDonald, M. D. Do Gulf toadfish use pulsatile urea excretion to chemically communicate reproductive status? *Physiol. Biochem. Zool.* **92**, 125-139 (2019).

32 Charlton, B. D. Discrimination of sex and reproductive state in koalas, Phascolarctos cinereus, using chemical cues in urine. *Anim. Behav.* **91**, 119-125 (2014).

33 Chase, R. & Blanchard, K. C. The snail's love-dart delivers mucus to increase paternity. *Proc. R. Soc. B: Biol. Sci.* **273**, 1471-1475 (2006).

34 Chasnov, J. R., So, W., Chan, C. & Chow, K. L. The species, sex, and stage specificity of a Caenorhabditis sex pheromone. *Proc. Natl. Acad. Sci. U.S.A.* **104**, 6730-6735 (2007).

35 Coffin, H. R., Watters, J. V. & Mateo, J. M. Odor-based recognition of familiar and related conspecifics: a first test conducted on captive Humboldt penguins (Spheniscus humboldti). *PloS One* **6**, e25002 (2011).

36 Coll, J. *et al.* Chemical aspects of mass spawning in corals. I. Sperm-attractant molecules in the eggs of the scleractinian coral Montipora digitata. *Mar. Biol.* **118**, 177-182 (1994).

37 Coll, J. *et al.* Chemical aspects of mass spawning in corals. II.(-)-Epi-thunbergol, the sperm attractant in the eggs of the soft coral Lobophytum crassum (Cnidaria: Octocorallia). *Mar. Biol.* **123**, 137-143 (1995).

38 Colyer, S. W. & Jenkins, C. Pheromonal control of aggressive display in Siamese fighting fish (Betta splendens). *Percept. Mot. Skills* **42**, 47-54 (1976).

39 Conner, W. E., Eisner, T., Vander Meer, R. K., Guerrero, A. & Meinwald, J. Precopulatory sexual interaction in an arctiid moth (Utetheisa ornatrix): role of a pheromone derived from dietary alkaloids. *Behav. Ecol. Sociobiol.* **9**, 227-235 (1981).

40 Cooper Jr, W. E. Chemosensory recognition of familiar and unfamiliar conspecifics by the scincid lizard Eumeces laticeps. *Ethology* **102**, 454-464 (1996).

41 Cosson, J., Carré, D. & Cosson, M. P. Sperm chemotaxis in siphonophores: identification and biochemical properties of the attractant. *Cell Motil. Cytoskelet.* **6**, 225-228 (1986).

42 Cummins, S. F. *et al.* Extreme aggression in male squid induced by a β-MSP-like pheromone. *Curr. Biol.* **21**, 322-327 (2011).

43 Dias, J. M., Segovia, J. M. & Willemart, R. H. Detection of conspecifics through olfaction in the Neotropical harvestman Mischonyx cuspidatus (Arachnida: Opiliones). *J. Arachnol.* **48**, 94-97 (2020).

44 Diaz, E. R. & Thiel, M. Chemical and visual communication during mate searching in rock shrimp. *Biol. Bull.* **206**, 134-143 (2004).

45 Douglas, H. D. Prenuptial perfume: alloanointing in the social rituals of the crested auklet (Aethia cristatella) and the transfer of arthropod deterrents. *Naturwissenschaften* **95**, 45-53 (2008).

46 Drickamer, L. C. Oestrous female house mice discriminate dominant from subordinate males and sons of dominant from sons of subordinate males by odour cues. *Anim. Behav.* (1992).

47 Eliott, S., Tait, N. & Briscof, D. A pheromonal function for the crural glands of the onychophoran Cephalofovea tomahmontis (Onychophora: Peripatopsidae). *J. Zool.* **231**, 1-9 (1993).

48 Evans, J. P., Garcia-Gonzalez, F., Almbro, M., Robinson, O. & Fitzpatrick, J. L. Assessing the potential for egg chemoattractants to mediate sexual selection in a broadcast spawning marine invertebrate. *Proc. R. Soc. B: Biol. Sci.* **279**, 2855-2861 (2012).

49 Eveland, L., Fried, B. & Cohen, L. Schistosoma mansoni: adult worm chemoattraction, with and without barriers. *Exp. Parasitol.* **54**, 271-276 (1982).

50 Fatsini, E. *et al.* Olfactory sensitivity of the marine flatfish Solea senegalensis to conspecific body fluids. *J. Exp. Biol.* **220**, 2057-2065 (2017).

51 Fernandes, N. S., Stanley, E., Costa, F. G., Toscano-Gadea, C. A. & Willemart, R. H. Chemical sex recognition in the harvestman Discocyrtus prospicuus (Arachnida: Opiliones). *Acta Ethol.* **20**, 215-221 (2017).

52 Fisher, H. S. & Rosenthal, G. G. Female swordtail fish use chemical cues to select well-fed mates. *Anim. Behav.* **72**, 721-725 (2006).

53 Fisher, H. S., Swaisgood, R. & Fitch-Snyder, H. Countermarking by male pygmy lorises (Nycticebus pygmaeus): do females use odor cues to select mates with high competitive ability? *Behav. Ecol. Sociobiol.* **53**, 123-130 (2003).

54 Fissette, S. D., Bussy, U., Huerta, B., Buchinger, T. J. & Li, W. Evidence that male sea lamprey increase pheromone release after perceiving a competitor. *J. Exp. Biol.* **223**, jeb226647 (2020).

55 Forsberg, L. A., Dannewitz, J., Petersson, E. & Grahn, M. Influence of genetic dissimilarity in the reproductive success and mate choice of brown trout–females fishing for optimal MHC dissimilarity. *J. Evol. Biol.* **20**, 1859-1869 (2007).

56 Franklin, A. M., Marshall, N. J. & Lewis, S. M. Multimodal signals: ultraviolet reflectance and chemical cues in stomatopod agonistic encounters. *Roy. Soc. Open Sci.* **3**, 160329 (2016).

57 Fried, B. & Haseeb, M. Intra-and interspecific chemoattraction in Echinostoma caproni and E. trivolvis adults in vitro. *J. Helminthol. Soc. Wash.* **57**, 72-73 (1990).

58 Fujimoto, Y., Yambe, H., Takahashi, K. & Sato, S. Bile from reproductively mature male largemouth bass Micropterus salmoides attracts conspecific females and offers a practical application to control populations. *Manag. Biol. Invasions* **11**, 415 (2020).

59 Fukakusa, C. K. Behavioral evidence of chemical communication by male caudal fin organs of a glandulocaudine fish (Teleostei: Characidae). *Ichthyol. Explor. Freshw.* **30**, 237-247 (2021).

60 Galtsoff, P. S. Physiology of reproduction of Ostrea virginica: II. Stimulation of spawning in the female oyster. *Biol. Bull.* **75**, 286-307 (1938).

61 García, L., Torrado-León, E., Talarico, G. & Peretti, A. V. First characterization of the behavioral repertory in a ricinuleid: Cryptocellus narino Platnick & Paz 1979 (Arachnida, Ricinulei, Ricinoididae). *J. Insect Behav.* **28**, 447-459 (2015).

62 Garm, A., Lebouvier, M. & Tolunay, D. Mating in the box jellyfish C opula sivickisi—Novel function of cnidocytes. *J. Morphol.* **276**, 1055-1064 (2015).

63 Gerlach, G. & Lysiak, N. Kin recognition and inbreeding avoidance in zebrafish, Danio rerio, is based on phenotype matching. *Anim. Behav.* **71**, 1371-1377 (2006).

64 Gonzalo, A., Cabido, C., Martín, J. & López, P. Detection and discrimination of conspecific scents by the anguid slow-worm Anguis fragilis. *J. Chem. Ecol.* **30**, 1565-1573 (2004).

65 Gorbman, A., Whiteley, A. & Kavanaugh, S. Pheromonal stimulation of spawning release of gametes by gonadotropin releasing hormone in the chiton, Mopalia sp. *Gen. Comp. Endocrinol.* **131**, 62-65 (2003).

66 Gosling, L. & McKay, H. Competitor assessment by scent matching: an experimental test. *Behav. Ecol. Sociobiol.* **26**, 415-420 (1990).

67 Grillet, M., Dartevelle, L. & Ferveur, J.-F. A Drosophila male pheromone affects female sexual receptivity. *Proc. R. Soc. B: Biol. Sci.* **273**, 315-323 (2006).

68 Hagelin, J. C. The citrus-like scent of crested auklets: reviewing the evidence for an avian olfactory ornament. *J. Ornithol.* **148**, 195 (2007).

69 Hardege, J., Bentley, M., Beckmann, M. & Müller, C. Sex pheromones in marine polychaetes: volatile organic substances (VOS) isolated from Arenicola marina. *Mar. Ecol. Prog. Ser.* **139**, 157-166 (1996).

70 Harris, R., Holland, B., Cameron, E., Davies, N. & Nicol, S. Chemical signals in the echidna: differences between seasons, sexes, individuals and gland types. *J. Zool.* **293**, 171-180 (2014).

71 Harris, R. L., Cameron, E. Z. & Nicol, S. C. A field study of wild echidna responses to conspecific odour. In: Buesching, C. (ed) *Chemical Signals in Vertebrates 14*. Springer, Nature Switzerland. p71-80 (2019)

72 Hawes, M. L. Odor as a possible isolating mechanism in sympatric species of shrews (Sorex vagrans and Sorex obscurus). *J. Mammal.* **57**, 404-406 (1976).

73 Heuschele, J. & Kiørboe, T. The smell of virgins: mating status of females affects male swimming behaviour in Oithona davisae. *J. Plankton Res.* **34**, 929-935 (2012).

74 Hicks, R. E., Larned, A. & Borgia, G. Bower paint removal leads to reduced female visits, suggesting bower paint functions as a chemical signal. *Anim. Behav.* **85**, 1209-1215 (2013).

75 Hirao, A., Aoyama, M. & Sugita, S. The role of uropygial gland on sexual behavior in domestic chicken Gallus gallus domesticus. *Behav. Process.* **80**, 115-120 (2009).

76 Hoppe, W. & Reichert, M. Predictable annual mass release of gametes by the coral reef sponge Neofibularia nolitangere (Porifera: Demospongiae). *Mar. Biol.* **94**, 277-285 (1987).

77 Hurst, J. L. The priming effects of urine substrate marks on interactions between male house mice, Mus musculus domesticus Schwarz & Schwarz. *Anim. Behav.* **45**, 55-81 (1993).

78 Hussain, Y. H., Sadilek, M., Salad, S., Zimmer, R. K. & Riffell, J. A. Individual female differences in chemoattractant production change the scale of sea urchin gamete interactions. *Develop. Biol.* **422**, 186-197 (2017).

79 Ibáñez, A., López, P. & Martín, J. Discrimination of conspecifics’ chemicals may allow Spanish terrapins to find better partners and avoid competitors. *Anim. Behav.* **83**, 1107-1113 (2012).

80 Ibáñez, A., Marzal, A., López, P. & Martín, J. Chemosensory assessment of rival body size is based on chemosignal concentration in male Spanish terrapins. *Behav. Ecol. Sociobiol.* **68**, 2005-2012 (2014).

81 Ingvarsdóttir, A. *et al.* Role of semiochemicals in mate location by parasitic sea louse, Lepeophtheirus salmonis. *J. Chem. Ecol.* **28**, 2107-2117 (2002).

82 Iyengar, V. K., Rossini, C. & Eisner, T. Precopulatory assessment of male quality in an arctiid moth (Utetheisa ornatrix): hydroxydanaidal is the only criterion of choice. *Behav. Ecol. Sociobiol.* **49**, 283-288 (2001).

83 Jacob, J., Balthazart, J. & Schoffeniels, E. Sex differences in the chemical composition of uropygial gland waxes in domestic ducks. *Biochem. Syst. Ecol.* **7**, 149-153 (1979).

84 Jacquemin, G. & Bareth, C. Ultrastructure des soies glandulaires et sensorielles du premier sternite abdominal des males de Campodea chardardi Condé (Insecta: Diplura): modifications liées à la mue. *Int. J. Insect Morphol. Embryol.* **10**, 463-481 (1981).

85 Jaeger, R. G., Goy, J. M., Tarver, M. & Márquez, C. E. Salamander territoriality: pheromonal markers as advertisement by males. *Anim. Behav.* **34**, 860-864 (1986).

86 Jenkins, H. L., Bishop, J. D. & Hughes, R. N. Prudent female allocation by modular hermaphrodites: female investment is promoted by the opportunity to outcross in cyclostome bryozoans. *Biol. J. Linn. Soc.* **116**, 593-602 (2015).

87 Johannesson, K. *et al.* Male discrimination of female mucous trails permits assortative mating in a marine snail species. *Evolution* **62**, 3178-3184 (2008).

88 Jones, G. *et al.* The worm has turned: Behavioural drivers of reproductive isolation between cryptic lineages. *Soil Biol. Biochem.* **98**, 11-17 (2016).

89 Jones, T. M. & Hamilton, J. A role for pheromones in mate choice in a lekking sandfly. *Anim. Behav.* **56**, 891-898 (1998).

90 Joss, J. M. The Australian lungfish, Neoceratodus forsteri: a personal story. *Gen. Comp. Endocrinol.* **173**, 1-3 (2011).

91 Kampfraath, A. A., Dudink, T. P., Kraaijeveld, K., Ellers, J. & Zizzari, Z. V. Male sexual trait decay in two asexual springtail populations follows neutral mutation accumulation Theory. *Evol. Biol.* **47**, 285-292 (2020).

92 Kasumyan, A. & Mamedov, C. A. Behavioral response of mature males of Acipenseridae to female sex pheromone. *J. Ichthyol.* **51**, 457-465 (2011).

93 Kasurak, A., Zielinski, B. S. & Higgs, D. M. Reproductive status influences multisensory integration responses in female round gobies, Neogobius melanostomus. *Anim. Behav.* **83**, 1179-1185 (2012).

94 Kavaliers, M. & Colwell, D. D. Discrimination by female mice between the odours of parasitized and non-parasitized males. *Proc. R. Soc. B: Biol. Sci.* **261**, 31-35 (1995).

95 Kearn, G. & Whittington, I. Sperm transfer in monogenean (platyhelminth) parasites. *Acta Parasitol.* **60**, 567-600 (2015).

96 Kekäläinen, J. *et al.* The information content of odour, colour and tactile cues in the mate choice of minnows. *Behaviour* **148**, 909-925 (2011).

97 Kelly, L. S., Snell, T. W. & Lonsdale, D. J. Chemical communication during mating of the harpacticoid Tigriopus japonicus. *Philos. Trans. R. Soc. B: Biol. Sci* **353**, 737-744 (1998).

98 Kholodnyy, V., Dzyuba, B., Gadêlha, H., Cosson, J. & Boryshpolets, S. Egg-sperm interaction in sturgeon: role of ovarian fluid. *Fish Physiol. Biochem.*, 1-17 (2020).

99 King, J. D., Rollins-Smith, L. A., Nielsen, P. F., John, A. & Conlon, J. M. Characterization of a peptide from skin secretions of male specimens of the frog, Leptodactylus fallax that stimulates aggression in male frogs. *Peptides* **26**, 597-601 (2005).

100 Klauser, M. D. Mucous secretions of the acoel turbellarian Convoluta sp. Ørsted: an ecological and functional approach. *J. Exp. Mar. Biol. and Ecol.* **97**, 123-133 (1986).

101 Koh, T. H., Seah, W. K., Yap, L.-M. Y. & Li, D. Pheromone-based female mate choice and its effect on reproductive investment in a spitting spider. *Behav. Ecol. Sociobiol.* **63**, 923-930 (2009).

102 Kortet, R. & Hedrick, A. The scent of dominance: female field crickets use odour to predict the outcome of male competition. *Behav. Ecol. Sociobiol.* **59**, 77-83 (2005).

103 Kosman, E. T., Hipp, B. & Levitan, D. R. Chemoattractant-mediated preference of non-self eggs in Ciona robusta sperm. *Biol. Bull.* **233**, 183-189 (2017).

104 Krueger, S., Moritz, G., Lindemann, P., Radisch, D. & Tschuch, G. Male pheromones influence the mating behavior of Echinothrips americanus. *J. Chem. Ecol.* **42**, 294-299 (2016).

105 Labra, A. Chemoreception and the assessment of fighting abilities in the lizard Liolaemus monticola. *Ethology* **112**, 993-999 (2006).

106 Lagoutte, R. *et al.* Total synthesis, proof of absolute configuration, and biosynthetic origin of stylopsal, the first isolated sex pheromone of Strepsiptera. *Chem. Eur. J.* **19**, 8515-8524 (2013).

107 Lambert, J. & Resink, J. Steroid glucuronides as male pheromones in the reproduction of the African catfish Clarias gariepinus—a brief review. *J. Steroid Biochem. Mol. Biol.* **40**, 549-556 (1991).

108 Landry, C., Garant, D., Duchesne, P. & Bernatchez, L. ‘Good genes as heterozygosity’: the major histocompatibility complex and mate choice in Atlantic salmon (Salmo salar). *Proc. R. Soc. B: Biol. Sci.* **268**, 1279-1285 (2001).

109 Leclaire, S., Strandh, M., Mardon, J., Westerdahl, H. & Bonadonna, F. Odour-based discrimination of similarity at the major histocompatibility complex in birds. *Proc. R. Soc. B: Biol. Sci.* **284**, 20162466 (2017).

110 LeMaster, M. P. & Mason, R. T. Variation in a female sexual attractiveness pheromone controls male mate choice in garter snakes. *J. Chem. Ecol.* **28**, 1269-1285 (2002).

111 Leonard, J. L. & Lukowiak, K. Courtship, copulation, and sperm trading in the sea slug, Navanax inermis (Opisthobranchia: Cephalaspidea). *Can. J. Zool.* **63**, 2719-2729 (1985).

112 Lin, C. Y., Tung, C. H., Yu, J. K. & Su, Y. H. Reproductive periodicity, spawning induction, and larval metamorphosis of the hemichordate acorn worm Ptychodera flava. *J. Exp. Zool. B Mol. Dev. Evol.* **326**, 47-60 (2016).

113 Littlewood, P. & Blower, J. The chemosensory behaviour of Lithobius forficatus. 1. Evidence for a pheromone released by the coxal organs (Myriapoda: Chilopoda). *J. Zool.* **211**, 65-82 (1987).

114 Locatello, L., Mazzoldi, C. & Rasotto, M. Ejaculate of sneaker males is pheromonally inconspicuous in the black goby, Gobius niger (Teleostei, Gobiidae). *J. Exp. Zool.* **293**, 601-605 (2002).

115 Lombardo, R. C. & Goshima, S. Female copulatory status and male mate choice in Neptunea arthritica (Gastropoda: Buccinidae). *J. Molluscan Stud.* **76**, 317-322 (2010).

116 López, P., Aragón, P. & Martín, J. Responses of female lizards, Lacerta monticola, to males' chemical cues reflect their mating preference for older males. *Behav. Ecol. Sociobiol.* **55**, 73-79 (2003).

117 Lüscher, A. & Wedekind, C. Size-dependent discrimination of mating partners in the simultaneous hermaphroditic cestode Schistocephalus solidus. *Behav. Ecol.* **13**, 254-259 (2002).

118 Ma, P. W. & Ramaswamy, S. B. Biology and ultrastructure of sex pheromone-producing tissue. In: Blomquist, G. & R. Vogt (eds) *Insect Biochemistry and Moecular Biology.* Elsevier. p19-51 (2003).

119 Madin, L. Sensory ecology of salps (Tunicata, Thaliacea): more questions than answers. *Mar. Freshw. Behav. Physiol.* **26**, 175-195 (1995).

120 Marchionni, V. & Rolando, A. Sex reversal in Ophryotrocha puerilis puerilis (Polychaeta, Dorvilleidae) induced by ethereal extracts of female phase individuals. *Ital. J. Zool.* **48**, 91-96 (1981).

121 Marco, A., Chivers, D. P., Kiesecker, J. M. & Blaustein, A. R. Mate choice by chemical cues in western redback (Plethodon vehiculum) and Dunn's (P. dunni) salamanders. *Ethology* **104**, 781-788 (1998).

122 Marneweck, C., Jürgens, A. & Shrader, A. Ritualised dung kicking by white rhino males amplifies olfactory signals but reduces odour duration. *J. Chem. Ecol.* **44**, 875-885 (2018).

123 Marneweck, C., Jürgens, A. & Shrader, A. M. Dung odours signal sex, age, territorial and oestrous state in white rhinos. *Proc. R. Soc. B: Biol. Sci.* **284**, 20162376 (2017).

124 Marneweck, C., Jürgens, A. & Shrader, A. M. The role of middens in white rhino olfactory communication. *Anim. Behav.* **140**, 7-18 (2018).

125 Marquet, N., Hubbard, P. C., da Silva, J. P., Afonso, J. & Canário, A. V. Chemicals released by male sea cucumber mediate aggregation and spawning behaviours. *Sci. Rep.* **8**, 1-13 (2018).

126 Martín, J. & López, P. Scent may signal fighting ability in male Iberian rock lizards. *Biol. Lett.* **3**, 125-127 (2007).

127 Martín, J. & López, P. Pheromones and reproduction in reptiles. In: Norris, D.O. & K.H. Lopez (eds) *Hormones and Reproduction of Vertebrates*. p141-167. Elsevier (2011).

128 Martín, J., Moreira, P. & López, P. Status‐signalling chemical badges in male Iberian rock lizards. *Funct. Ecol.* **21**, 568-576 (2007).

129 Mathews, L. M. Tests of the mate-guarding hypothesis for social monogamy: male snapping shrimp prefer to associate with high-value females. *Behav. Ecol.* **14**, 63-67 (2003).

130 Mathis, A. Territorial salamanders assess sexual and competitive information using chemical signals. *Anim. Behav.* **40**, 953-962 (1990).

131 Matsumura, K. Tetrodotoxin as a pheromone. *Nature* **378**, 563-564 (1995).

132 Mau, A., Bingham, J.-P., Soller, F. & Jha, R. Maturation, spawning, and larval development in captive yellowfoot limpets (Cellana sandwicensis). *Invert. Reprod. Develop.* **62**, 239-247 (2018).

133 McDonough, C. M. Pairing behavior of the nine-banded armadillo (Dasypus novemcinctus). *Am. Midl. Nat.*, 290-298 (1997).

134 Mehr, S. *et al.* Transcriptome sequencing and annotation of the polychaete Hermodice carunculata (Annelida, Amphinomidae). *BMC Genom.* **16**, 1-13 (2015).

135 Milinski, M. *et al.* Mate choice decisions of stickleback females predictably modified by MHC peptide ligands. *Proc. Natl. Acad. Sci. U.S.A.* **102**, 4414-4418 (2005).

136 Miller, A. L. & Formanowicz, D. R. Friend or foe: behavioral responses to conspecifics in the northern scorpion, Paruroctonus boreus (Scorpionida: Vaejovidae). *J. Ethol.* **29**, 251-256 (2011).

137 Miller, R. Sperm chemotaxis in the hydromedusae. I. Species-specificity and sperm behavior. *Mar. Biol.* **53**, 99-113 (1979).

138 Miller, R. L. Demonstration of sperm chemotaxis in echinodermata: Asteroidea, Holothuroidea, Ophiuroidea. *J. Exp. Zool.* **234**, 383-414 (1985).

139 Miller, R. L. Evidence for the presence of sexual pheromones in free-spawning starfish. *J. Exp. Mar. Biol. Ecol.* **130**, 205-221 (1989).

140 Miller, R. L. & King, K. R. Sperm chemotaxis in Oikopleura dioica fol, 1872 (Urochordata: Larvacea). *Biol. Bull.* **165**, 419-428 (1983).

141 Mohebbi, G. *et al.* Acetylcholinesterase inhibitory activity of a neurosteroidal alkaloid from the upside-down jellyfish Cassiopea andromeda venom. *Rev. Bras. Farmacogn.* **28**, 568-574 (2018).

142 Moomjian, L., Nystrom, S. & Rittschof, D. Behavioral responses of sexually active mud snails: kariomones and pheromones. *J. Chem. Ecol.* **29**, 497-501 (2003).

143 Moore, A. J. & Moore, P. J. Balancing sexual selection through opposing mate choice and male competition. *Proc. R. Soc. B: Biol. Sci.* **266**, 711-716 (1999).

144 Moore, P. J., Reagan-Wallin, N. L., Haynes, K. F. & Moore, A. J. Odour conveys status on cockroaches. *Nature* **389**, 25-25 (1997).

145 Morse, P., Zenger, K. R., McCormick, M. I., Meekan, M. G. & Huffard, C. L. Chemical cues correlate with agonistic behaviour and female mate choice in the southern blue-ringed octopus, Hapalochlaena maculosa (Hoyle, 1883)(Cephalopoda: Octopodidae). *J. Molluscan Stud.* **83**, 79-87 (2017).

146 Müller, G. Über Gordiaceen. *Zeitschrift für Morphologie und Ökologie der Tiere* **7**, 134-219 (1926).

147 Muñoz-Romo, M., Burgos, J. F. & Kunz, T. H. Smearing behaviour of male Leptonycteris curasoae (Chiroptera) and female responses to the odour of dorsal patches. *Behaviour*, 461-483 (2011).

148 Nakashima, Y. Mucous trail following in 2 intertidal nudibranchs. *J. Ethol.* **13**, 125-128 (1995).

149 Nhan, H. T., Jung, L. H., Ambak, M. A., Watson, G. J. & Siang, H. Y. Evidence for sexual attraction pheromones released by male tropical donkey's ear abalone (Haliotis asinina),(L.). *Invert. Reprod. Develop.* **54**, 169-176 (2010).

150 Noer, C. L., Balsby, T. J. S., Anistoroaei, R., Stelvig, M. & Dabelsteen, T. Mate choice screening in captive solitary carnivores: The role of male behavior and cues on mate preference and paternity in females of a model species, American mink (Neovison vison). *Zoo Biol.* **36**, 367-381 (2017).

151 Novo, M., Riesgo, A., Fernández-Guerra, A. & Giribet, G. Pheromone evolution, reproductive genes, and comparative transcriptomics in Mediterranean earthworms (Annelida, Oligochaeta, Hormogastridae). *Mol. Biol. Evolut.* **30**, 1614-1629 (2013).

152 Painter, S. D. *et al.* Relative contributions of the egg layer and egg cordon to pheromonal attraction and the induction of mating and egg-laying behavior in Aplysia. *Biol. Bull.* **181**, 81-94 (1991).

153 Palumbi, S. R. All males are not created equal: fertility differences depend on gamete recognition polymorphisms in sea urchins. *Proc. Natl. Acad. Sci. U.S.A.* **96**, 12632-12637 (1999).

154 Parrott, M. L., Ward, S. J. & Temple-Smith, P. D. Genetic similarity, not male size, influences female mate choice in the agile antechinus (Antechinus agilis). *Aust. J. Zool.* **54**, 319-323 (2006).

155 Parrott, M. L., Ward, S. J. & Temple-Smith, P. D. Olfactory cues, genetic relatedness and female mate choice in the agile antechinus (Antechinus agilis). *Behav. Ecol. Sociobiol.* **61**, 1075-1079 (2007).

156 Patlar, B., Weber, M., Temizyürek, T. & Ramm, S. A. Seminal fluid-mediated manipulation of post-mating behavior in a simultaneous hermaphrodite. *Curr. Biol.* **30**, 143-149. e144 (2020).

157 Pietsch, T. W. Dimorphism, parasitism, and sex revisited: modes of reproduction among deep-sea ceratioid anglerfishes (Teleostei: Lophiiformes). *Ichthyol. Res.* **52**, 207-236 (2005).

158 Porco, D., Deharveng, L. & Skarżyński, D. Sex pheromone in Tetrodontophora bielanesis (Waga, 1842)(Collembola: Onychiuridae): Indirect reproduction mediated by cuticular compounds. *Pedobiologia* **53**, 59-63 (2009).

159 Rajagopal, T., Archunan, G., Geraldine, P. & Balasundaram, C. Assessment of dominance hierarchy through urine scent marking and its chemical constituents in male blackbuck Antelope cervicapra, a critically endangered species. *Behav. Process.* **85**, 58-67 (2010).

160 Ram, J. L., Fong, P. P. & Garton, D. W. Physiological aspects of zebra mussel reproduction: maturation, spawning, and fertilization. *Am. Zool.* **36**, 326-338 (1996).

161 Rantala, M. J., Jokinen, I., Kortet, R., Vainikka, A. & Suhonen, J. Do pheromones reveal male immunocompetence? *Proc. R. Soc. B: Biol. Sci.* **269**, 1681-1685 (2002).

162 Rasmussen, L. & Schulte, B. Chemical signals in the reproduction of Asian (Elephas maximus) and African (Loxodonta africana) elephants. *Anim. Reprod. Sci.* **53**, 19-34 (1998).

163 Rasmy, A. & Hussein, H. Effect of age and mating on release of female sex pheromones and male response in the two‐spotted spider mite. *J. Appl. Entomol.* **117**, 109-111 (1994).

164 Reece-Engel, C. Female choice of resident male rabbits Oryctolagus cuniculus. *Anim. Behav.* (1988).

165 Reusch, T. B., HaÈberli, M. A., Aeschlimann, P. B. & Milinski, M. Female sticklebacks count alleles in a strategy of sexual selection explaining MHC polymorphism. *Nature* **414**, 300-302 (2001).

166 Riesgo, A., Farrar, N., Windsor, P. J., Giribet, G. & Leys, S. P. The analysis of eight transcriptomes from all poriferan classes reveals surprising genetic complexity in sponges. *Mol. Biol. Evolut.* **31**, 1102-1120 (2014).

167 Roberts, J. A. & Uetz, G. W. Information content of female chemical signals in the wolf spider, Schizocosa ocreata: male discrimination of reproductive state and receptivity. *Anim. Behav.* **70**, 217-223 (2005).

168 Roe, P. & Norenburg, J. L. Morphology and taxonomic distribution of a newly discovered feature, postero-lateral glands, in pelagic nemerteans. *Hydrobiologia* **456**, 133-144 (2001).

169 Rogers, D. C. Mate-searching behaviour and sex pheromones in Branchinecta lindahli Packard, 1883 (Branchiopoda: Anostraca). *J. Crust. Biol.* **39**, 1-10 (2019).

170 Romero-Lebrón, E., Oviedo‐Diego, M. A., Elias, D., Vrech, D. E. & Peretti, A. V. Effect of the mating plug on female chemical attractiveness and mating acceptance in a scorpion. *Ethology* **125**, 184-194 (2019).

171 Roth, O., Sundin, J., Berglund, A., Rosenqvist, G. & Wegner, K. Male mate choice relies on major histocompatibility complex class I in a sex‐role‐reversed pipefish. *J. Evol. Biol.* **27**, 929-938 (2014).

172 Run, J.-Q., Chen, C.-P., Chang, K.-H. & Chia, F.-S. Mating behaviour and reproductive cycle of Archaster typicus (Echinodermata: Asteroidea). *Mar. Biol.* **99**, 247-253 (1988).

173 Santer, R. D. & Hebets, E. A. The sensory and behavioural biology of whip spiders (Arachnida, Amblypygi). Adv. Insect. Physiol **41,**1–64. (2011)

174 Sasson, D. A., Jacquez, A. A. & Ryan, J. F. The ctenophore Mnemiopsis leidyi regulates egg production via conspecific communication. *BMC Ecol.* **18**, 1-10 (2018).

175 Saunders, K. M., Brockmann, H. J., Watson III, W. H. & Jury, S. H. Male horseshoe crabs Limulus polyphemus use multiple sensory cues to locate mates. *Curr. Zool.* **56**, 485-498 (2010).

176 Schaller, F. Indirect sperm transfer by soil arthropods. *Annu. Rev. Entomol.* **16**, 407-446 (1971).

177 Schjørring, S. Sex allocation and mate choice of selfed and outcrossed Schistocephalus solidus (Cestoda). *Behav. Ecol.* **20**, 644-650 (2009).

178 Schjørring, S. & Jäger, I. Incestuous mate preference by a simultaneous hermaphrodite with strong inbreeding depression. *Evolution* **61**, 423-430 (2007).

179 Schmit, O. *et al.* Mate recognition as a reproductive barrier in sexual and parthenogenetic Eucypris virens (Crustacea, Ostracoda). *Anim. Behav.* **85**, 977-985 (2013).

180 Schneeberger, K., Schulze, M., Scheffler, I. & Caspers, B. A. Evidence of female preference for odor of distant over local males in a bat with female dispersal. *Behav. Ecol.* **32**, 657-661 (2021).

181 Schneider, R. A. Z., Huber, R. & Moore, P. Individual and status recognition in the crayfish, Orconectes rusticus: the effects of urine release on fight dynamics. *Behaviour* **138**, 137-153 (2001).

182 Schnier, J. *et al.* Ultrastructure of the epidermal gland system of Tetranchyroderma suecicum Boaden, 1960 (Gastrotricha: Macrodasyida) indicates a defensive function of its exudate. *Zoomorphology* **138**, 443-462 (2019).

183 Schubert, M., Rödel, H. G., Pillay, N. & von Holst, D. Promiscuous tendencies in the round-eared sengi: a male’s perspective. *J. Ethol.* **30**, 43-51 (2012).

184 Snell, T. W., Kim, J., Zelaya, E. & Resop, R. Mate choice and sexual conflict in Brachionus plicatilis (Rotifera). *Hydrobiologia* **593**, 151-157 (2007).

185 Sombke, A. & Müller, C. H. When SEM becomes a deceptive tool of analysis: the unexpected discovery of epidermal glands with stalked ducts on the ultimate legs of geophilomorph centipedes. *Front. Zool.* **18**, 1-19 (2021).

186 Sonenshine, D. E. Tick pheromones and their use in tick control. *Annu. Rev. Entomol.* **51**, 557-580 (2006).

187 Soong, K., Chang, D. & Chao, S. Presence of spawn-inducing pheromones in two brittle stars (Echinodermata: Ophiuroidea). *Mar. Ecol. Prog. Ser.* **292**, 195-201 (2005).

188 Sorensen, P. & Winn, H. The induction of maturation and ovulation in American eels, Anguilla rostrata (LeSueur), and the relevance of chemical and visual cues to male spawning behaviour. *J. Fish Biol.* **25**, 261-268 (1984).

189 Stoddart, D. M. & Fairall, N. Electrocardiographic technique for studying olfactory response in the rock hyrax Procavia capensis L.(mammalia: hyracoidea). *J. Chem. Ecol.* **7**, 257-263 (1981).

190 Strandh, M. *et al.* Major histocompatibility complex class II compatibility, but not class I, predicts mate choice in a bird with highly developed olfaction. *Proc. R. Soc. B: Biol. Sci.* **279**, 4457-4463 (2012).

191 Tapia, C. *et al.* Courtship behavior and potential indications for chemical communication in Artemia franciscana (Kellog 1906)/Conducta de cortejo y potenciales indicadores de comunicacion quimica en Artemia franciscana (Kellog 1906). *Gayana* **79**, 152 (2015).

192 Taylor, A. *et al.* A Sperm Spawn-Inducing Pheromone in the Silver Lip Pearl Oyster (Pinctada maxima). *Mar. Biotechnol.* **20**, 531-541 (2018).

193 Thornhill, R. Female preference for the pheromone of males with low fluctuating asymmetry in the Japanese scorpionfly (Panorpa japonica: Mecoptera). *Behav. Ecol.* **3**, 277-283 (1992).

194 Thünken, T., Bakker, T. C. & Baldauf, S. A. “Armpit effect” in an African cichlid fish: self-referent kin recognition in mating decisions of male Pelvicachromis taeniatus. *Behav. Ecol. Sociobiol.* **68**, 99-104 (2014).

195 Thünken, T. *et al.* Size-related inbreeding preference and competitiveness in male Pelvicachromis taeniatus (Cichlidae). *Behav. Ecol.* **22**, 358-362 (2011).

196 Townsend, C. R. Mucus trail following by the snail Biomphalaria glabrata (Say). *Anim. Behav.* **22**, 170-177 (1974).

197 Trouvé, S. & Coustau, C. Differences in adult excretory-secretory products between geographical isolates of Echinostoma caproni. *J. Parasitol.*, 1062-1065 (1998).

198 Trouvé, S., Renaud, F., Durand, P. & Jourdane, J. Selfing and outcrossing in a parasitic hermaphrodite helminth (Trematoda, Echinostomatidae). *Heredity* **77**, 1-8 (1996).

199 Tsuneki, K., Suzuki, A. & Ouji, M. Sex difference in the cloacal gland in the hagfish, Eptatretus burgeri, and its possible significance in reproduction. *Acta Zool.* **66**, 151-158 (1985).

200 Twan, W.-H., Hwang, J.-S. & Chang, C.-F. Sex steroids in scleractinian coral, Euphyllia ancora: implication in mass spawning. *Biol. Reprod.* **68**, 2255-2260 (2003).

201 Van Damme, K. & Dumont, H. J. Sex in a cyclical parthenogen: mating behaviour of Chydorus sphaericus (Crustacea; Branchiopoda; Anomopoda). *Freshw. Biol.* **51**, 2334-2346 (2006).

202 Van Leeuwen, H. & Maly, E. Changes in swimming behavior of male Diaptomus leptopus (Copepoda: Calanoida) in response to gravid females. *Limnol. Oceanogr.* **36**, 1188-1195 (1991).

203 Wagenaar, D. A., Hamilton, M. S., Huang, T., Kristan, W. B. & French, K. A. A hormone-activated central pattern generator for courtship. *Curr. Biol.* **20**, 487-495 (2010).

204 Walls, S. C., Mathis, A., Jaeger, R. G. & Gergits, W. F. Male salamanders with high-quality diets have faeces attractive to females. *Anim. Behav.* **38**, 546-548 (1989).

205 Wanlong, Z., Fangyan, Y. & Zhengkun, W. Study of chemical communication based on urine in tree shrews Tupaia belangeri (Mammalia: Scandentia: Tupaiidae). *Eur. Zool. J.* **84**, 512-524 (2017).

206 Warbeck, A. & Parzefall, J. Mate recognition via waterborne chemical cues in the viviparous caecilian Typhlonectes natans (Amphibia: Gymnophiona). In: Marchlewska-Koj, A., J. L. Lepri, & D. Müller-Schwarze (eds) *Chemical Signals in Vertebrates 9.* p263–268 (2001)

207 Ward, G. E., Brokaw, C. J., Garbers, D. L. & Vacquier, V. D. Chemotaxis of Arbacia punctulata spermatozoa to resact, a peptide from the egg jelly layer. *J. Cell Biol.* **101**, 2324-2329 (1985).

208 Watson, G., Langford, F., Gaudron, S. & Bentley, M. Factors influencing spawning and pairing in the scale worm Harmothoe imbricata (Annelida: Polychaeta). *Biol. Bull.* **199**, 50-58 (2000).

209 Wedekind, C., Seebeck, T., Bettens, F. & Paepke, A. J. MHC-dependent mate preferences in humans. *Proc. R. Soc. B: Biol. Sci.* **260**, 245-249 (1995).

210 Westermann, B. & Beuerlein, K. Y-maze experiments on the chemotactic behaviour of the tetrabranchiate cephalopod Nautilus pompilius (Mollusca). *Mar. Biol.* **147**, 145-151 (2005).

211 Weygoldt, P. Vergleichende untersuchungen zur Fortpflanzungsbiologie der pseudoscorpione. *Zeitschrift für Morphologie und Ökologie der Tiere* **56**, 39-92 (1966).

212 Wharton, R. A. Biology of the diurnal Metasolpuga picta (Kraepelin)(Solifugae, Solpugidae) compared with that of nocturnal species. *J. Arachnol.* **14**, 363-383 (1986).

213 Willemart, R. & Hebets, E. Sexual differences in the behavior of the harvestman Leiobunum vittatum (Opiliones, Sclerosomatidae) towards conspecific cues. *J. Insect Behav.* **25**, 12-23 (2012).

214 Willemart, R. H., Farine, J. P. & Gnaspini, P. Sensory biology of Phalangida harvestmen (Arachnida, Opiliones): a review, with new morphological data on 18 species. *Acta Zool.* **90**, 209-227 (2009).

215 Zhang, J.-X., Wei, W., Zhang, J.-H. & Yang, W.-H. Uropygial gland-secreted alkanols contribute to olfactory sex signals in budgerigars. *Chem. Senses* **35**, 375-382 (2010).

216 Ziegelmann, B., Lindenmayer, A., Steidle, J. & Rosenkranz, P. The mating behavior of Varroa destructor is triggered by a female sex pheromone. *Apidologie* **44**, 314-323 (2013).

217 Ziegelmann, B., Tolasch, T., Steidle, J. L. & Rosenkranz, P. The mating behavior of Varroa destructor is triggered by a female sex pheromone. Part 2: Identification and dose-dependent effects of components of the Varroa sex pheromone. *Apidologie* **44**, 481-490 (2013).

218 Zizzari, Z. V., Braakhuis, A., van Straalen, N. M. & Ellers, J. Female preference and fitness benefits of mate choice in a species with dissociated sperm transfer. *Anim. Behav.* **78**, 1261-1267 (2009).

219 Zizzari, Z. V. *et al.* Love at first sniff: a spermatophore-associated pheromone mediates partner attraction in a collembolan species. *Anim. Behav.* **124**, 221-227 (2017).

220 Zuri, I., Su, W. & Halpern, M. Conspecific odor investigation by gray short-tailed opossums (Monodelphis domestica). *Physiol. Behav.* **80**, 225-232 (2003).

221 Charlton, B. D. Chemosensory discrimination of identity and familiarity in koalas. *Behav. Process.* **119**, 38-43 (2015).

222 Alvarino, A. Chaetognatha. In Adiyodi, K. G. & R. G. Adiyodi (eds) *Reproductive Biology of Invertebrates. Vol 5. Sexual Differentiation and Behaviour.* John Wiley & Sons, Chichester. pp. 425–470 (1992)

223 Aizen, J., Meiri, I., Tzchori, I., Levavi-Sivan, B. & Rosenfeld, H. Enhancing spawning in the grey mullet (Mugil cephalus) by removal of dopaminergic inhibition. *Gen. Comp. Endocrinol.* **142**, 212-221 (2005).

224 Alcock, J. The Scramble Competition Mating System of the Dark Fishfly (Nigronia serricornis)(Megaloptera: Corydalidae). *Northeast. Nat.* **21**, 351-356 (2014).

225 Aldrich, J. R. & Zhang, Q.-H. Chemical ecology of Neuroptera. *Annu. Rev. Entomol.* **61**, 197-218 (2016).

226 Bandaranayake, W. M., Bourne, D. J. & Sim, R. G. Chemical composition during maturing and spawning of the sponge Dysidea herbacea (Porifera: Demospongiae). *Comp. Biochem. Physiol. B, Biochem. Mol. Biol.* **118**, 851-859 (1997).

227 Bandilla, M., Hakalahti-Sirén, T. & Valtonen, E. Experimental evidence for a hierarchy of mate-and host-induced cues in a fish ectoparasite, Argulus coregoni (Crustacea: Branchiura). *Int. J. Parasitol.* **37**, 1343-1349 (2007).

228 Basaran, F., Muhtaroglu, C. G., Özden, O. & Özkizilcik, S. Spawning behaviour of shi drum (Umbrina cirrosa) after hormone administration. *J. Fish. Sci.* **3**, 124 (2009).

229 Belda, C. & Del Norte, A. Notes on the induced spawning and larval rearing of the Asian moon scallop, Amusium pleuronectes (Linne), in the laboratory. *Aquaculture* **72**, 173-179 (1988).

230 Bhatnagar, K. & Wible, J. Observations on the vomeronasal organ of the colugo Cynocephalus (Mammalia, Dermoptera). *Cells Tissues Organs* **151**, 43-48 (1994).

231 Bi‐song, W., Fang‐dong, Z., Qi‐zhi, S. & Jing, L. Mating behavioor of the cat flea, Ctenocephaslides felis bouche (Siphonaptera: Pulicidae) and male responses to female extract on an artificial feeding system. *Insect Sci.* **9**, 29-34 (2002).

232 Hardege, J.D. Nereidid polychaetes as model organisms for marine chemical ecology. *Hydrobiologia.* **402**, 145-161 (1999).

233 Bonner, T. P. & Etges, F. J. Chemically mediated sexual attraction in Trichinella spiralis. *Exp. Parasitol.* **21**, 53-60 (1967).

234 Boxshall, G. A. On the anatomy of the misophrioid copepods, with special reference to Benthomisophria palliata Sars. *Philos. Trans. R. Soc. B: Biol. Sci* **297**, 125-181 (1982).

235 Boxshall, G. A. & Lincoln, R. J. The life cycle of the Tantulocarida (Crustacea). *Philos. Trans. R. Soc. B: Biol. Sci* **315**, 267-303 (1987).

236 Brown, R. Armadillos, sloths, anteaters and pangolins: orders Edentata and Pholidotain. In: Brown R.E. & D.W. Macdonald (eds) *Social Odours in Mammals Vol. 2.* Oxford University Press. p732-738 (1985).

237 Burke, N. W., Crean, A. J. & Bonduriansky, R. The role of sexual conflict in the evolution of facultative parthenogenesis: a study on the spiny leaf stick insect. *Anim. Behav.* **101**, 117-127 (2015).

238 Byrnes, G., Lim, N. T.-L., Yeong, C. & Spence, A. J. Sex differences in the locomotor ecology of a gliding mammal, the Malayan colugo (Galeopterus variegatus). *J. Mammal.* **92**, 444-451 (2011).

239 Campbell, A., Coppard, S., D’abreo, C. & Tudor-Thomas, R. Escape and aggregation responses of three echinoderms to conspecific stimuli. *Biol. Bull.* **201**, 175-185 (2001).

240 Castro, I. *et al.* Olfaction in birds: a closer look at the kiwi (Apterygidae). *J. Avian Biol.* **41**, 213-218 (2010).

241 Chapman, D. D., Corcoran, M. J., Harvey, G. M., Malan, S. & Shivji, M. S. Mating behavior of southern stingrays, Dasyatis americana (Dasyatidae). *Environ. Biol. Fishes* **68**, 241-245 (2003).

242 Chauhan, K. R., Levi, V., Zhang, Q.-H. & Aldrich, J. R. Female goldeneyed lacewings (Neuroptera: Chrysopidae) approach but seldom enter traps baited with the male-produced compound iridodial. *J. Econ. Entomol.* **100**, 1751-1755 (2007).

243 Chiussi, R. & DÍaz, H. A laboratory study on the visual and chemical orientation of the gastropod Nerita fulgurans Gmelin, 1791. *Mar. Freshw. Behav. Physiol.* **35**, 167-177 (2002).

244 Chung, M. L. S., Galano, J.-M., Oger, C., Durand, T. & Lee, J. C.-Y. Hyperoxia elevates adrenic acid peroxidation in marine fish and is associated with reproductive pheromone mediators. *Mar. Drugs* **13**, 2215-2232 (2015).

245 Crespo, J. G. A review of chemosensation and related behavior in aquatic insects. *J. Insect Sci.* **11** (2011).

246 Cuzin-Roudy, J. Reproduction in northern krill (Meganyctiphanes norvegica Sars). In Tarling, G.A. (ed) *Advances in Marine Biology* *Vol. 57.* P199-230 (2010).

247 Dallai, R., Mercati, D., Mashimo, Y., Machida, R. & Beutel, R. The morphology and ultrastructure of salivary glands of Zoraptera (Insecta). *Arthropod Struct. Dev.* **46**, 508-517 (2017).

248 DiPaola, J. D., Yindee, M. & Plotnik, J. M. Investigating the use of sensory information to detect and track prey by the Sunda pangolin (Manis javanica) with conservation in mind. *Sci. Rep.* **10**, 1-10 (2020).

249 Duncan, T. K. Sexual dimorphism and reproductive behavior in Almyracuma proximoculi (Crustacea: Cumacea): the effect of habitat. *Biol. Bull.* **165**, 370-378 (1983).

250 Eberhard, M. J. & Picker, M. D. Vibrational communication in two sympatric species of Mantophasmatodea (Heelwalkers). *J. Insect Behav.* **21**, 240-257 (2008).

251 Eisenberg, J. F. & Kleiman, D. G. Olfactory communication in mammals. *Ann. Rev. Ecol. Syst.* **3**, 1-32 (1972).

252 Farnkopf, I. C. *et al.* Olfactory epithelium and ontogeny of the nasal chambers in the bowhead whale (Balaena mysticetus). *Anat. Rec.* **305**, 643-667 (2022).

253 Garcia-Rejon, L., Sanchez-Moreno, M., Verdejo, S. & Monteoliva, M. Site of sex pheromone production in Ascaris suum (Nematoda). *Can. J. Zool.* **63**, 664-665 (1985).

254 Goenaga, C. *Two New Species of Stichopathes (Zoantharia; Antipatharia) with Observations on Aspects of their Biology*, M.Sc. Thesis, University of Puerto Rico (1977).

255 Gordon, I. Pre-copulatory behaviour of captive sandtiger sharks, Carcharias taurus. In: Demski L.S. & J.P. Wourms (eds) *The Reproduction and Development of Sharks, Skates, Rays and Ratfishes.* Springer Dordrecht. P159-164 (1993).

256 Haney, T. A. & Martin, J. W. A new genus and species of leptostracan (Crustacea: Malacostraca: Phyllocarida) from Guana Island, British Virgin Islands, and a review of leptostracan genera. *J. Nat. Hist.* **38**, 447-469 (2004).

257 Haszprunar, G. The fine morphology of the osphradial sense organs of the Mollusca. IV. Caudofoveata and Solenogastres. *Philos. Trans. R. Soc. B: Biol. Sci* **315**, 63-73 (1987).

258 Haszprunar, G. The fine morphology of the osphradial sense organs of the Mollusca. III. Placophora and Bivalvia. *Philos. Trans. R. Soc. B: Biol. Sci* **315**, 37-61 (1987).

259 Hirano, Y. & Inaba, A. Siphonaria (pulmonate limpet) survey of Japan-I. Observations on the behavior of Siphonaria japonica during breeding season. *Publ. Seto Mar. Biol. Lab.* **25**, 323-334 (1980).

260 Hoch, J. M., Schneck, D. T. & Neufeld, C. J. Ecology and evolution of phenotypic plasticity in the penis and cirri of barnacles. *Integr. Comp. Biol.* **56**, 728-740 (2016).

261 Holland, N. D. & Grimmer, J. C. Epidermal mucus and the reproduction of a crinoid echinoderm. *Nature* **255**, 223-224 (1975).

262 Hsueh, Y.-P., Leighton, D. H. & Sternberg, P. W. Nematode communication. In Witzany G. (ed) *Biocommunication of Animals.* Springer Dordrecht. 383-407 (2014).

263 Hubbard, P. C., Barata, E. N. & Canário, A. V. Olfactory sensitivity of the gilthead seabream (Sparus auratus L) to conspecific body fluids. *J. Chem. Ecol.* **29**, 2481-2498 (2003).

264 Iliffe, T. M. & Pearse, J. S. Annual and lunar reproductive rhythms of the sea urchin, Diadema antillarum (Philippi) in Bermuda. *Int. J. Invertebr. Reprod.* **5**, 139-148 (1982).

265 Johansson, K. U. & Hallberg, E. Male-specific structures in the olfactory system of mysids (Mysidacea; Crustacea). *Cell Tissue Res.* **268**, 359-368 (1992).

266 Johnson, C. & Johnson, K. Behaviour of the bilby, Macrotis lagotis (Reid),(Marsupialia: Thylacomyidae) in captivity. *Wildl. Res.* **10**, 77-87 (1983).

267 Johnson, R. H. & Nelson, D. R. Copulation and possible olfaction-mediated pair formation in two species of carcharhinid sharks. *Copeia* **1978**, 539-542 (1978).

268 Joshi, M. *The Role of Male Chemical Secretion Components in Sex Recognition, Mate Assessment and Mate Xhoice in the Diurnal Gecko Cnemaspis mysoriensis*. M.Sc. Thesis, Indian Institute of Science Education and Research (2020).

269 Joshi, M., Ellsworth, B. & Thaker, M. Single components of complex chemical signals convey sex identity and individual variation. *Anim. Behav.* **187**, 1-13 (2022).

270 Jumper Jr, G. Y. & Baird, R. C. Location by olfaction: a model and application to the mating problem in the deep-sea hatchetfish Argyropelecus hemigymnus. *Am. Nat.* **138**, 1431-1458 (1991).

271 Kajiura, S. M., Sebastian, A. P. & Tricas, T. C. Dermal bite wounds as indicators of reproductive seasonality and behaviour in the Atlantic stingray, Dasyatis sabina. *Environ. Biol. Fishes* **58**, 23-31 (2000).

272 Kenning, M., Müller, C. H. & Sombke, A. The ultimate legs of Chilopoda (Myriapoda): a review on their morphological disparity and functional variability. *PeerJ* **5**, e4023 (2017).

273 Kowalski, R. *et al.* Semen biology and stimulation of milt production in the European smelt (Osmerus eperlanus L.). *Aquaculture* **261**, 760-770 (2006).

274 Lam, T. Artificial propagation of milkfish: present status and problems. In: *Advances in Milkfish Biology and Culture: Proceedings of the Second International Milkfish Aquaculture Conference, 4-8 October 1983, Iloilo City, Philippines.* Aquaculture Department, Southeast Asian Fisheries Development Center. p21-39 (1984).

275 Larsen, K., Guţu, M. & Sieg, J. Order Tanaidacea Dana, 1849. In: von Vaupel Klein, J.C. (ed) *Treatise on Zoology-Anatomy, Taxonomy, Biology. The Crustacea, Volume 5.* Brill, p249-329 (2015).

276 Legg, G. Taxonomy and the dangers of sex with special reference to Pseudoscorpions. *Adv. Arachnol. Dev. Biol* **14**, 247-257 (2009).

277 Lindberg, D. R. & Ponder, W. F. The influence of classification on the evolutionary interpretation of structure a re-evaluation of the evolution of the pallial cavity of gastropod molluscs. *Org. Divers. Evol.* **1**, 273-299 (2001).

278 Luer, C. A. & Gilbert, P. W. Mating behavior, egg deposition, incubation period, and hatching in the clearnose skate, Raja eglanteria. *Environ. Biol. Fishes* **13**, 161-171 (1985).

279 MacKinnon, B. Sex attractants in nematodes. *Parasitol. Today* **3**, 156-158 (1987).

280 Margulies, D. *et al.* Spawning and early development of captive yellowfin tuna (Thunnus albacares). *Fish. Bull.* **105** (2007).

281 Marshall, D. J., Semmens, D. & Cook, C. Consequences of spawning at low tide: limited gamete dispersal for a rockpool anemone. *Mar. Ecol. Prog. Ser.* **266**, 135-142 (2004).

282 Martin, R. A. A review of behavioural ecology of whale sharks (Rhincodon typus). *Fish. Res.* **84**, 10-16 (2007).

283 Matsumoto, S., Takeyama, T., Ohnishi, N. & Kohda, M. Mating system and size advantage of male mating in the protogynous swamp eel Monopterus albus with paternal care. *Zool. Sci.* **28**, 360-367 (2011).

284 McEuen, F. Spawning behaviors of northeast Pacific sea cucumbers (Holothuroidea: Echinodermata). *Mar. Biol.* **98**, 565-585 (1988).

285 Mercier, A. & Hamel, J.F. Endogenous and exogenous control of gametogenesis and spawning in echinoderms. Adv. Mar. Biol. **55** p1-302 (2009).

286 Middleton, H. *Pheromone Utilisation in Elasmobranchs*. Ph.D. Dissertation, The University of Queensland (2022).

287 Mies, M. & Sumida, P. Y. Giant clam aquaculture: a review on induced spawning and larval rearing. *Int. J. Mar. Sci.* **2** (2012).

288 Miller, L. C. *Aspects of the Ecology and Sociobiology of the Parasite Moniliformis dubius (Acanthocephala)*. Ph.D. dissertation, New Mexico State University (1980).

289 Mori, N. & Kuwahara, Y. Comparative studies of the ability of males to discriminate between sexes in Caloglyphus spp. *J. Chem. Ecol.* **26**, 1299-1309 (2000).

290 Munn, E., Klepal, W. & Barnes, H. The fine structure and possible function of the sensory setae of the penis of Balanus balanoides (L.). *J. Exp. Mar. Biol. Ecol.* **14**, 89-98 (1974).

291 Nikitin, M. Bioinformatic prediction of Trichoplax adhaerens regulatory peptides. *Gen. Comp. Endocrinol.* **212**, 145-155 (2015).

292 Obst, M. & Funch, P. Dwarf male of Symbion pandora (Cycliophora). *J. Morphol.* **255**, 261-278 (2003).

293 Poduschka, W. Insectivore communication. In: T.A. Sebeok (ed) *How Animals Communicate.* Indiana University Press, Bloomington IN. p600-633 (1977).

294 Potier, S. *et al.* Preen oil chemical composition encodes individuality, seasonal variation and kinship in black kites Milvus migrans. *J. Avian Biol.* **49**, e01728 (2018).

295 Quarrell, S. R. *et al.* Identification of the putative aggregation pheromone components emitted by the European earwig, Forficula auricularia. *Chemoecology* **26**, 173-186 (2016).

296 Reber, S. A. Crocodilians Are Promising Intermediate Model Organisms for Comparative Perception Research. *Comp. Cogn. Behav. Rev.* **15** (2020).

297 Rebora, M., Piersanti, S., Frati, F. & Salerno, G. Antennal responses to volatile organic compounds in a stonefly. *J. Insect Physiol.* **98**, 231-237 (2017).

298 Reynierse, J. H. & Gleason, K. Determinants of planarian aggregation behavior. *Anim. Learn. Behav.* **3**, 343-346 (1975).

299 Grieves, L. A. *et al.* Olfactory camouflage and communication in birds. Biol. Rev. Camb. Philos. Soc. **97**, 1193-1209 (2022).

300 Rivers, T. J. & Morin, J. G. Plasticity of male mating behaviour in a marine bioluminescent ostracod in both time and space. *Anim. Behav.* **78**, 723-734 (2009).

301 Rohner, C. A. *et al.* Population ecology of whale sharks. In: Dove A.D.M. & S.J. Pierce (eds) *Whale Sharks: Biology, Ecology, and Conservation.* CRC Press, Boca Raton, FL. p129-1152 (2021).

302 Salamon, M., Davies, N. W. & Stoddart, D. M. Olfactory communication in Australian marsupials with particular reference to Brushtail possum, koala, and eastern grey kangaroo. In: Johnston R.E., D. Müller-Schwarze, & P.W. Sorensen (eds) *Advances in Chemical Signals in Vertebrates.* Springer New York, NY. p85-98 (1999).

303 Schlötzer-Schrehardt, U. Ultrastructural investigation of the nuchal organs of Pygospio elegans (Polychaeta) II. Adult nuchal and dorsal organs. *Zoomorphology* **107**, 169-179 (1987).

304 Shao, Y., Lu, Y. & Wei, Z. Study on methods of extraction and bioassay test sex pheromone of adult female of psocids Liposcelis entomophila. *Acta Ecol. Sin.* **26**, 2148-2153 (2006).

305 Shinya, R., Chen, A. & Sternberg, P. W. Sex attraction and mating in Bursaphelenchus okinawaensis and B. xylophilus. *J. Nematol.* **47**, 176 (2015).

306 Slattery, M., Hines, G., Starmer, J. & Paul, V. Chemical signals in gametogenesis, spawning, and larval settlement and defense of the soft coral Sinularia polydactyla. *Coral Reefs* **18**, 75-84 (1999).

307 Stender-Seidel, S., Thomas, G. & Böckeler, W. Investigation of different ontogenetic stages of Raillietiella sp.(Pentastomida: Cephalobaenida): accessory genital glands. *Parasitol. Res.* **83**, 264-268 (1997).

308 Suárez-Morales, E. Monstrilloid copepods: the best of three worlds. *Bull. South. Calif. Acad. Sci.* **117**, 92-103 (2018).

309 Syrnikov, J. S., Melnitsky, S. I. & Ivanov, V. D. Effect of feeding to the mating behaviour of Trichoptera. In: Tanida K. & A. Rossiter (eds) *Proceedings of the 11th International Symposium on Trichoptera.* Tokai University Press, Kanagawa. p413-419 (2005).

310 Tanabe, T. & Sota, T. Complex copulatory behavior and the proximate effect of genital and body size differences on mechanical reproductive isolation in the millipede genus Parafontaria. *Am. Nat.* **171**, 692-699 (2008).

311 Thiel, M. & Junoy, J. Mating behavior of nemerteans: present knowledge and future directions. *J. Nat. Hist.* **40**, 1021-1034 (2006).

312 Weeks, S. C. & Benvenuto, C. Mate guarding in the androdioecious clam shrimp Eulimnadia texana: male assessment of hermaphrodite receptivity. *Ethology* **114**, 64-74 (2008).

313 Wölper, C. Das osphradium der Paludina vivipara. *Zeitschrift für vergleichende Physiologie* **32**, 272-286 (1950).

314 Wongthamwanich, N. *et al.* Daily activities of the giant pill-millipede Zephronia cf. viridescens Attems, 1936 (Diplopoda: Sphaerotheriida: Zephroniidae) in a deciduous forest in northern Thailand. *Zool. Stud.* **51**, 913-926 (2012).

315 Wu, N. C. & Waas, J. R. No evidence for across-population scent discrimination of cloacal gland secretions in tuatara (Sphenodon punctatus). *J. Herpetol.* **51**, 178-185 (2017).

316 Young, C., Tyler, P., Cameron, J. & Rumrill, S. Seasonal breeding aggregations in low-density populations of the bathyal echinoid Stylocidaris lineata. *Mar. Biol.* **113**, 603-612 (1992).

317 Grieves, L., Gloor, G., Bernards, M. & MacDougall-Shackleton, E. Songbirds show odour-based discrimination of similarity and diversity at the major histocompatibility complex. *Anim. Behav.* **158**, 131-138 (2019).

318 Leclaire, S. *et al.* An individual and a sex odor signature in kittiwakes? Study of the semiochemical composition of preen secretion and preen down feathers. *Naturwissenschaften* **98**, 615-624 (2011).

319 Whittaker, D. J. *et al.* Intraspecific preen oil odor preferences in dark-eyed juncos (Junco hyemalis). *Behav. Ecol.* **22**, 1256-1263 (2011).
